# Metal–Organic Frameworks Meet Two‐Dimensional Materials in Polymer Matrices for Flame Retardant and Sensor Applications

**DOI:** 10.1002/smsc.202400611

**Published:** 2025-02-21

**Authors:** Xue Bi, Yanan Hou, Ye‐Tang Pan, Siqi Huo, Congling Shi, Jiyu He, Rongjie Yang

**Affiliations:** ^1^ Beijing Key Laboratory of MetroFire and Passenger Transportation Safety China Academy of Safety Science andTechnology Beijing 100012 China; ^2^ National Engineering ResearchCenter of Flame Retardant Materials School of Materials Science & Engineering, Beijing Institute of Technology Beijing 100081 PR China; ^3^ College of Geological Engineeringand Geomatics Chang'an University Xi'an 710064 Shaanxi China; ^4^ School of Engineering, Centre for Future Materials University of Southern Queensland Springfield 4300 Australia

**Keywords:** flame retardant, metal–organic frameworks, performance and mechanism, sensing, two‐dimensional materials

## Abstract

Functional polymer composites offer versatility and high performance through material fusion, but flammability is an obstacle to application. Metal–organic frameworks (MOFs) have attracted attention in the field of flame retardant due to their structural diversity and high specific surface area, but they suffer from low efficiency and agglomeration issues when used alone. Combining with two‐dimensional (2D) nanomaterials can improve the above situation. Herein, strategies are explored for integrating MOFs with 2D materials through physical mixing and in situ growth to enhance their dispersion and flame‐retardant effects in polymers. Additionally, the integration of sensing performance can achieve intelligent monitoring and control, as well as real‐time risk assessment and system optimization. In summary, this review deeply analyzes the dispersion, interfacial interaction, and performance adjustment mechanism of composite materials and discusses in detail the application potential of MOFs and hybrids formed by 2D materials in the field of flame retardant and sensing. Finally, the opportunities and challenges faced by the integration of MOFs and 2D materials in functional polymer composites in the future are summarized and prospected. Herein, it is also expected to facilitate researchers to quickly understand the latest developments in the field and guide their effective design.

## Graphical abstract

Herein, strategies are examined for integrating metal–organic frameworks with two‐dimensional materials to enhance polymer flame retardancy and sensing properties, assessing their potential applications and outlining the opportunities and challenges of such integration in functional composites.

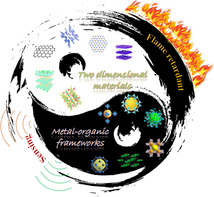

## 1 Introduction

In the pursuit of high‐performance and multifunctional materials, the preparation of functional polymer composites has emerged as a pivotal research frontier within the domain of materials science. These composites cater to the demands of contemporary society for versatile, high‐performance materials by ingeniously integrating materials with diverse properties, thereby achieving a blend of attributes that are unattainable with individual materials. They exhibit extensive potential across various sectors, including energy,^[^ ^1^, ^2^, ^3^ ^]^ environmental,^[^ ^4^, ^5^, ^6^ ^]^ medical,^[^ ^7^, ^8^, ^9^ ^]^ electronics,^[^ ^10^, ^11^, ^12^ ^]^ and aerospace^[^ ^13^, ^14^, ^15^ ^]^ and other fields.^[^ ^16^, ^17^, ^18^, ^19^, ^20^ ^]^ However, a significant challenge arises from their high flammability when exposed to open flames, posing a considerable threat to human life and property safety. Consequently, the development of materials possessing superior flame‐retardant properties is crucial in mitigating the risk of fire hazards.^[^ ^21^, ^22^, ^23^ ^]^

Flame‐retardant materials augment safety by attenuating the propagation of fire, thereby affording individuals extended temporal windows for evacuation and rescue operations while mitigating property damage and subsequent remedial expenditures. From an environmental standpoint, these materials adhere to the principles of sustainable development. Additionally, the integration of sensing properties into flame‐retardant materials facilitates intelligent monitoring and control mechanisms, enhancing system efficiency and reducing maintenance costs. Notably, in industries such as power generation and automotive manufacturing, these multifunctional materials enable real‐time risk evaluation and system optimization. Consequently, flame‐retardant materials possessing sensing capabilities confer substantial advantages in terms of safety, versatility, system performance, and maintenance, portending their potential for extensive application across diverse fields in the future.

Metal–organic frameworks (MOFs) have attracted much attention in the preparation of functional polymer composites due to their unique structure and properties. MOFs are composed of organic ligands and metal ions/clusters, exhibiting structural diversity and high specific surface area,^[^ ^24^ ^]^ and the representative types include ZIF, MIL and UiO.^[^ ^25^ ^]^ Its permanent porosity and high surface area make MOFs have a wide range of applications in gas separation,^[^ ^26^ ^]^ energy conversion and storage,^[^ ^27^ ^]^ heterogeneous catalysis,^[^ ^28^ ^]^ sensing,^[^ ^29^ ^]^ and biomedicine.^[^ ^30^ ^]^ Especially in the field of flame‐retardant polymer materials,^[^ ^21^, ^31^, ^32^, ^33^, ^34^, ^35^, ^36^ ^]^ MOFs are attracting attention for their transition metal elements. MOFs can adsorb toxic gases and improve the safety of flame‐retardant materials. Its porous structure and high thermal stability contribute to the formation of a char layer and slow down the rate of combustion. The organic part of MOFs enhances the compatibility of the filler with the polymer chain, which improves the dispersion of MOFs in the polymer matrix and further improves the flame retardant effect. MOFs are easy to synthesize and modify and can be chemically modified to introduce flame‐retardant groups to achieve customized designs to meet different polymer needs.

However, MOFs nanoparticles are susceptible to agglomeration within polymers, which adversely impacts not only their dispersibility within the polymer matrix but also the mechanical properties and flame retardancy of the polymer. Consequently, enhancing the dispersion of MOFs in polymers has emerged as a pivotal area of research. Present strategies to address this issue encompass modifying the surface functionality of MOFs nanoparticles to mitigate agglomeration and incorporate additional flame‐retardant elements. Alternatively, selecting an appropriate carrier, such as the introduction of a 2D material to uniformly disperse MOF nanoparticles, or directly utilizing MOF‐derived 2D materials, can augment flame retardancy while imparting versatility to the polymer. This approach aims to surmount the limitations of MOFs in terms of stability, catalysis, and dispersion, while integrating the distinctive advantages of 2D materials with the characteristics of MOFs, thereby broadening their application scope.

In the realm of nanomaterials, 2D nanomaterials distinguish themselves in flame retardancy owing to their layered architecture, effectively mitigating heat release rates and mass loss, and establishing a physical barrier. Consequently, they are extensively employed in flame‐resistant materials. Notable examples of 2D nano‐flame retardants encompass graphene and its derivatives,^[^ ^37^, ^38^, ^39^ ^]^ hexagonal boron nitride (h‐BN),^[^ ^40^, ^41^, ^42^ ^]^ MXene,^[^ ^43^, ^44^, ^45^ ^]^ covalent organic frameworks (COFs),^[^ ^46^, ^47^, ^48^ ^]^ MoS_2_,^[^ ^49^ ^]^ LDH, transition metal disulfides (TMDs),^[^ ^50^, ^51^, ^52^ ^]^ and layered metal phosphates. Despite the substantial performance of individual 2D materials, the construction of heterostructures and hybrid assemblies is pivotal for achieving tailored properties. Beyond their exceptional performance and the ease of modulating their chemical structure and surface characteristics, these 2D materials present a plethora of options for composite design.^[^ ^53^, ^54^, ^55^ ^]^ When MOFs are integrated with 2D materials, their combined synergies amplify respective strengths, yielding high‐performance functional polymer composites.^[^ ^56^, ^57^, ^58^, ^59^, ^60^, ^61^ ^]^ These composites amalgamate the high porosity and catalytic attributes of MOFs with the electrical and mechanical properties of 2D materials, rendering them an efficacious and dependable solution across various domains. Exploring the integration strategy of MOFs and 2D materials within functional polymer composites holds immense significance in advancing the field of materials science.

In this article, we discuss composite methods for MOFs and 2D materials through a variety of strategies, including but not limited to physical mixing, chemical bonding, and in situ growth. We conduct a detailed examination of the dispersion, interfacial interaction, and performance modulation mechanisms of these composites within the polymer matrix. The discussion is centered on their applications in two pivotal areas: enhancing flame retardant performance and developing sensor functionalities. Through structural design and functional integration, the key performance indicators of polymer materials, such as thermal stability, flame retardant efficacy, and sensing sensitivity, can be significantly improved. On this foundation, this article comprehensively analyzes the current research advancements in this field, summarizing successful cases and experiences while objectively highlighting the existing challenges and limitations. These include compatibility issues during compounding, difficulties in scaling up production, and assessments of long‐term stability.

## 2 MOFs and 2D Materials as Advanced Structures

### 2.1 MOFs

**Figure 1:**
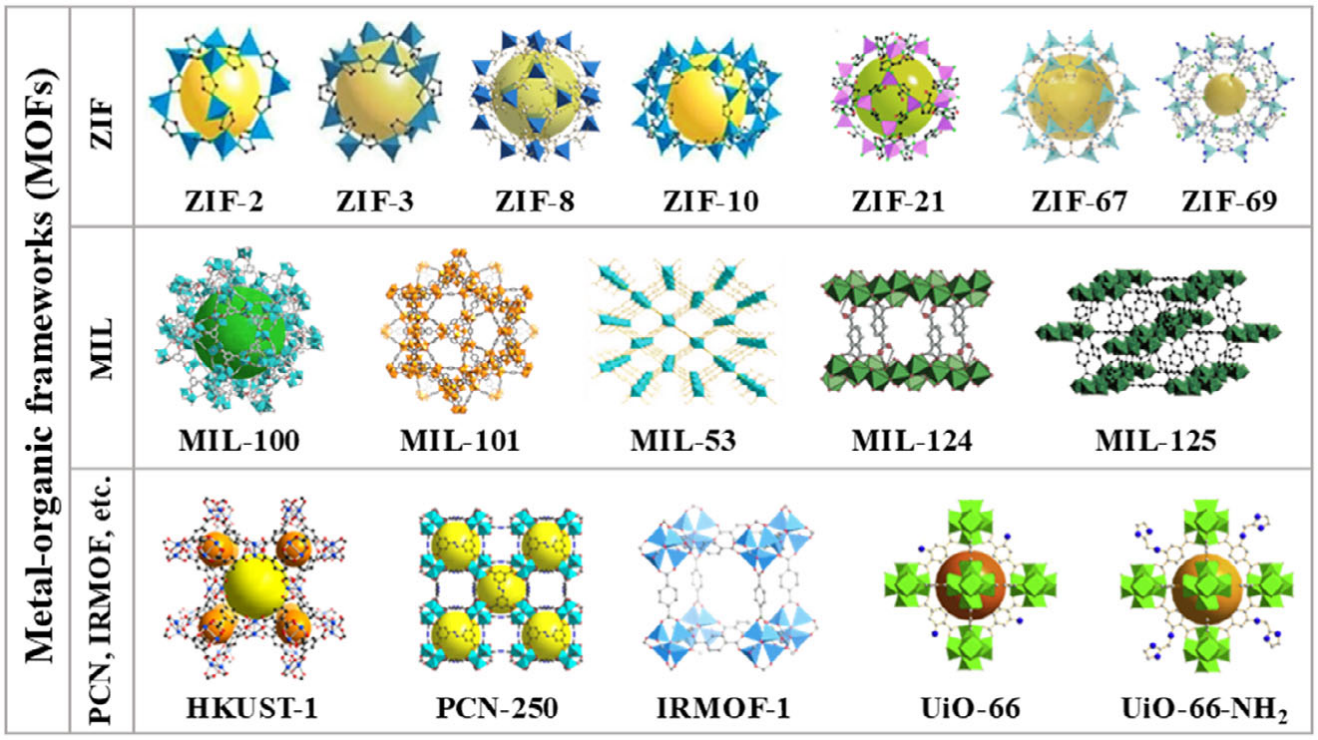
Crystal structure of representative MOFs (Reproduced (Adapted) with permission.^[^ ^104^ ^]^ Copyright 2022, John Wiley and Sons; Reproduced (Adapted) with permission.^[^ ^105^ ^]^ Copyright 2009, Royal Society of Chemistry; Reproduced (Adapted) with permission.^[^ ^106^ ^]^ Copyright 2024, Elsevier; Reproduced (Apadted) with permission.^[^ ^107^ ^]^ Copyright 2019, Elsevier; Reproduced (Adapted) with permission.^[^ ^108^ ^]^ Copyright 2021, Elsevier; Reproduced (Adapted) with permission.^[^ ^109^ ^]^ Copyright 2022, Elsevier; Reproduced (Adapted) with permission.^[^ ^110^ ^]^ Copyright 2021, Royal Society of Chemistry; Reproduced (Adapted) with permission.^[^ ^111^ ^]^ Copyright 2021, Elsevier).

MOFs are a class of crystalline porous materials with periodic network structure formed by the interconnection of inorganic metal centers (metal ions or metal clusters) and bridging organic ligands through self‐assembly (**Figure** **1**). MOFs are a type of organic–inorganic hybrid material, also known as coordination polymers. It differs from both inorganic porous materials and organic complexes in general. It has both the rigidity of inorganic materials and the flexibility of organic materials, which shows great development potential and remarkable development prospects in modern materials research.^[^ ^32^, ^62^, ^63^ ^]^

Compared with other traditional porous materials, MOFs exhibit significant advantages in terms of specific surface area, porosity, designability, and diversity, which underpin their functional versatility and broad application scope. Their ultrahigh specific surface area and favorable pore structure enable MOFs to accommodate guest molecules with substantial capacity and excellent selectivity, facilitating the storage of gases such as acetylene, hydrogen, and methane, as well as the capture of carbon dioxide. Furthermore, the tunability of their composition, structure, pore size, volume, ease of functionalization, flexible network, and accessible metal sites render MOFs advantageous for the adsorption and release of biomolecules.

MOFs have been extensively investigated in the context of heterogeneous catalysis, with a primary emphasis on metal nodes, framework nodes, the integration of homogeneous catalysts within the framework, molecular species encapsulated within MOFs, clusters confined by MOFs, and functionalized organic linkers or pores. The contribution of both inorganic and organic components to luminescence imparts MOFs with significant potential as multifunctional luminescent materials. The internal luminescence of MOFs, resulting from metal–ligand charge transfer, enhances their three‐dimensional luminescent capabilities. Zirconium‐based MOFs are distinguished by their diverse structures, stability, and favorable characteristics, rendering them suitable for practical applications. Additionally, researchers have explored the potential of MOFs in renewable energy and environmental applications, underscoring their remarkable porosity and tailorable chemical compositions. Consequently, MOFs emerge as versatile and potent materials with a broad spectrum of applications spanning gas storage and adsorption, catalysis, medicine, sensors, energy storage materials, among others.

### 2.2 Two‐Dimensional Materials

**Figure 2:**
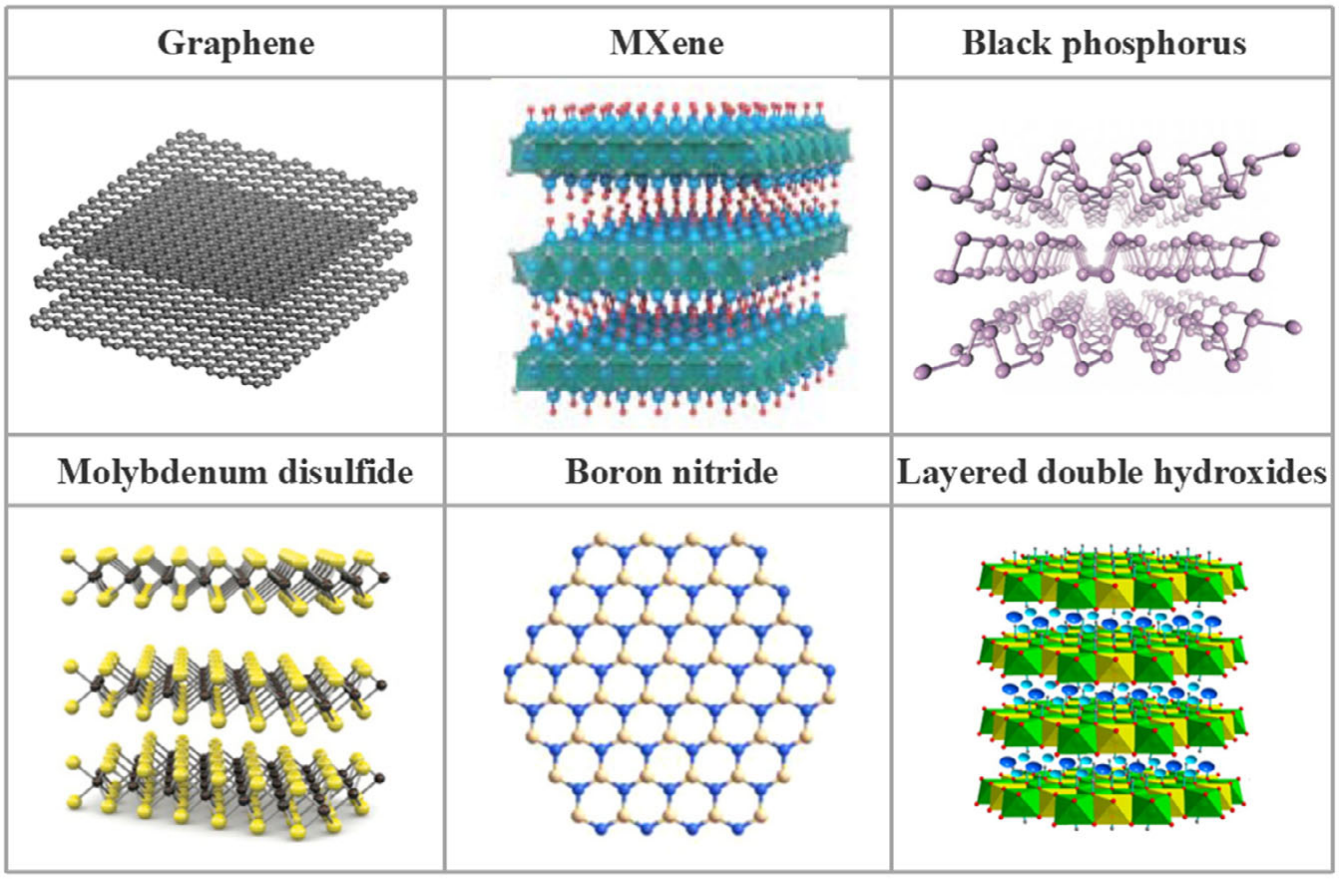
Schematic diagram of different kinds of typical 2D nanomaterials such as GO, MXene (Reproduced (Adapted) with permission. Copyright 2014, Springer Nature), BP (Reproduced (Adapted) with permission. Copyright 2021, Springer Nature), MoS_2_ (Reproduced (Adapted) with permission. Copyright 2016, Elsevier), h‐BN (Reproduced (Adapted) with permission. Copyright 2020, American Chemical Society), and LDH (Reproduced (Adapted) with permission. Copyright 2024, Elsevier).^[^ ^112^, ^113^, ^114^, ^115^, ^116^ ^]^

Since their emergence, 2D materials have garnered considerable attention across numerous scientific disciplines owing to their distinctive advantages and diverse classifications. The planar atoms in 2D materials are interconnected by robust covalent bonds, conferring a degree of flexibility and optical transparency. Concurrently, the electrons within these materials propagate within a 2D plane, manifesting exceptional electrical properties. Their reduced thickness and extensive surface area contribute to a substantial specific surface area. Furthermore, 2D materials encompass a wide range of electrical behaviors, spanning insulators, semiconductors, semimetals, metals, and superconductors. Notable examples of 2D materials include graphene oxide (GO), reduced graphene oxide (rGO), layered double hydroxides (LDH), MXenes, montmorillonite, mica flakes, molybdenum disulfide (MoS_2_), and black phosphorus (BP), among others (as depicted in **Figure** **2**). These materials are of particular interest due to their unique physical architectures and chemical attributes.

#### 2.2.1 Graphene

GO is a 2D crystalline material composed of a single layer of carbon atoms with a hexagonal honeycomb lattice structure. It is an important member of the carbon material family and has a close relationship with graphite, charcoal, carbon nanotubes, and fullerenes. The discovery and research of GO began in 2004 when two physicists from the University of Manchester in the UK successfully isolated a single layer of GO from graphite. The structure of GO is very stable, and the connections between carbon atoms are flexible and can be bent and deformed without rearrangement, a property that gives GO excellent thermal conductivity and electron transport properties. The electrons in GO move through orbit with little to no lattice defects or scattering by foreign atoms, which makes it extremely high in carrier mobility of 15 000 cm^2^ (V s)^−1^ at room temperature.

GO has shown great potential in many fields. In electronics, GO can be used to make smaller, lighter electronic components that improve the efficiency and performance of devices. In the biomedical field, GO can be used to make biosensors for detecting and monitoring living cells in biology. In addition, GO also has a wide range of application prospects in energy engineering, nanotechnology, electromagnetic shielding, and other fields.

#### 2.2.2 MXene

MXene is an emerging class of 2D inorganic compound materials. Since it was first reported by a research team at Drexel University in United States in 2011, it has attracted attention for its unique physicochemical properties and wide application potential. These materials are composed of transition metal carbides, nitrides, or carbonitrides of several atomic layers thick, with accordion‐like multilayer structures (ML‐MXene) or thin layer structures (FL‐MXene).

Excellent conductivity and pseudocapacitance are among the distinguishing features of MXene. This is due to its special structure of alternating carbon and transition metal layers. This structure not only provides more channels for the movement of ions, significantly increasing the speed of ion movement but also makes MXene have great potential for application in electrode materials. In addition, MXene exhibits wide‐range tunable IR radiation characteristics, as well as excellent optical properties and surface plasmon effects. These characteristics make it have a wide range of application prospects in many fields such as photothermal conversion, infrared identification/camouflage, surface plasmon, photocatalysis, and electrocatalysis. Interestingly, MXene is not only used as a stand‐alone material but also successfully integrated as MXene/polymer nanocomposites, MXene hydrogels, and MXene derived nanomaterials. Similarly, their synthesis and fabrication strategies, their chemistry and functionalization, and nano‐engineered structures provide MXene with a great platform for advanced nanomaterials.

#### 2.2.3 Boron Nitride

As an important inorganic nonmetallic material, BN has attracted extensive attention in the materials science community due to its unique physicochemical properties and wide range of application fields. BN exists in a variety of crystal forms, including hexagonal BN (h‐BN, also known as graphitic BN or white graphite) and cubic BN (c‐BN), which give BN diverse properties and application values. H‐BN has a layered structure similar to graphite. However, the nitrogen atoms and boron atoms are alternately arranged between the layers to form stable covalent bonds, which makes h‐BN have excellent lubricity, thermal stability, and chemical inertness. These characteristics make h‐BN have excellent stability in high temperature, high pressure, strong radiation, and corrosive environments and are widely used in high‐temperature lubricants, ceramic fillers, thermally conductive materials, and composite reinforcement phases. C‐BN is a superhard material synthesized by high temperature and high pressure, and its hardness is second only to diamond, and its thermal stability is better than that of diamond. Therefore, it is known as the “second hardest material.” With excellent wear resistance, high hardness, and good chemical stability, c‐BN is an ideal material for manufacturing high‐performance cutting tools, abrasives and wear‐resistant components. It is widely used in machining, geological exploration, and aerospace and other fields.

In addition, BN also has good electrical insulation and neutron absorption capacity, making it potentially valuable in electronic devices and nuclear reactor control materials. In recent years, with the rapid development of nanotechnology, boron nitride nanomaterials (such as BN nanotubes and BN nanosheets) have become one of the hot spots in materials science research due to their unique nano effects and excellent properties.

#### 2.2.4 Layered Double Hydroxide

LDH, also known as layered bimetallic hydroxide, is a class of inorganic crystalline materials with a special layered structure. They are comprised of multiple positively charged layers of metal hydroxides interspersed with anions that serve to balance the charge within the interlayer spaces. The interlayer anions and the interlayer forces are relatively weak, allowing for their ready exchange with other anions. This structural feature imparts LDHs with a high capacity for ion exchange and a controllable pore structure, thereby rendering them extensively utilized in applications such as adsorption, catalysis, controlled drug delivery, flame‐retardant materials, and other diverse fields.

The composition and structure of LDHs exhibit a remarkable degree of tunability. Their properties can be tailored by modifying the type and ratio of metal cations, the species of interlayer anions, and the content of interlayer water. Magnesium‐aluminum LDHs (Mg‐Al LDHs), for instance, are a prevalent type of LDHs that possess a structure analogous to hydrotalcite and exhibit superior adsorption properties and catalytic activity. In the context of adsorption, LDHs, owing to their highly controllable pore structure and surface‐active sites, demonstrate favorable adsorption characteristics towards specific organic compounds and ions, rendering them suitable for applications in wastewater treatment and gas separation, among others. In catalysis, LDHs serve as effective catalysts or catalyst supports and find widespread use in organic synthesis and catalytic conversions, such as oxidation reactions and esterification reactions. Furthermore, LDHs can also function as drug carriers, enabling controlled drug release and enhancing the bioavailability and therapeutic efficacy of pharmaceutical agents.

In recent years, with the rapid development of nanotechnology and materials science, remarkable progress has been made in the preparation and application of LDHs. LDHs with different structures and properties can be obtained through different preparation methods, such as co‐precipitation, ion exchange, and roasting and restoration, etc. These materials have demonstrated considerable potential for application in environmental remediation, energy conversion, biomedicine, and other domains, thereby presenting novel opportunities for advancements in science and technology as well as industrial development.

#### 2.2.5 Covalent Organic Framework

2D COFs are an emerging class of 2D porous polymers. Its structural characteristics are that the organic nodes are closely connected to the linker through covalent bonds, so as to construct a periodic porous structure with a high degree of order. These materials exhibit atomic‐scale thicknesses and long‐range ordered porous arrangements in 2D planes. In the out‐of‐plane direction, the structural stability of COFs is further enhanced by *π*−*π* stacking, and a series of unique physicochemical properties are endowed to COFs. These unique properties make COFs show broad application potential in many fields. From the perspective of material preparation, COFs materials are often used to prepare discontinuous films as well as partially crystalline films. Structurally, these films contain both the properties of amorphous materials and some of the orderliness of the crystal domain. This unique structural feature provides the possibility for the application of COFs materials in the fields of adsorption, separation, energy storage, and catalysis. In recent years, with the deepening of the research on COFs materials, COFs materials with specific pore structures have been synthesized. COFs are one of the potential candidates for high‐performance electrocatalysts. This discovery not only broadens the application range of COFs materials but also provides new ideas for their application in the field of energy conversion and storage. In summary, as a new type of porous organic material with unique structure and properties, COFs have broad application prospects in many fields.

#### 2.2.6 Other 2D Materials

MoS_2_ is an inorganic compound that is the main component of molybdenite. Its unique layered structure makes it easy to peel off from layer to layer, showing good lubricity and anisotropy. In addition to its excellent tribological properties, MoS_2_ is widely used in lubricants, friction materials, and coatings. Because of its semiconductor characteristics and band structure, it also shows great potential in electronic devices, optoelectronic devices, and energy storage. Especially at the nanoscale, single‐layer MoS_2_ exhibits direct bandgap characteristics, which makes it have a broad application prospect in the fields of transistors, photodetectors, and solar cells.

BP is a layered material formed by phosphorus atoms, and the layers are bound to each other by the weak van der Waals force. The layered structure of BP gives it unique electrical, optical, and mechanical properties. As a 2D semiconductor material, the energy bandgap of BP is adjustable in the range of 0.3–2.0 eV. It has potential applications in high‐performance electronics, optoelectronics, and field‐effect transistors. In addition, the layered structure of BP makes it an effective solid lubricant that can be used to reduce friction and wear.

## 3 Integration Strategy of MOFs with 2D Materials

**Figure 3:**
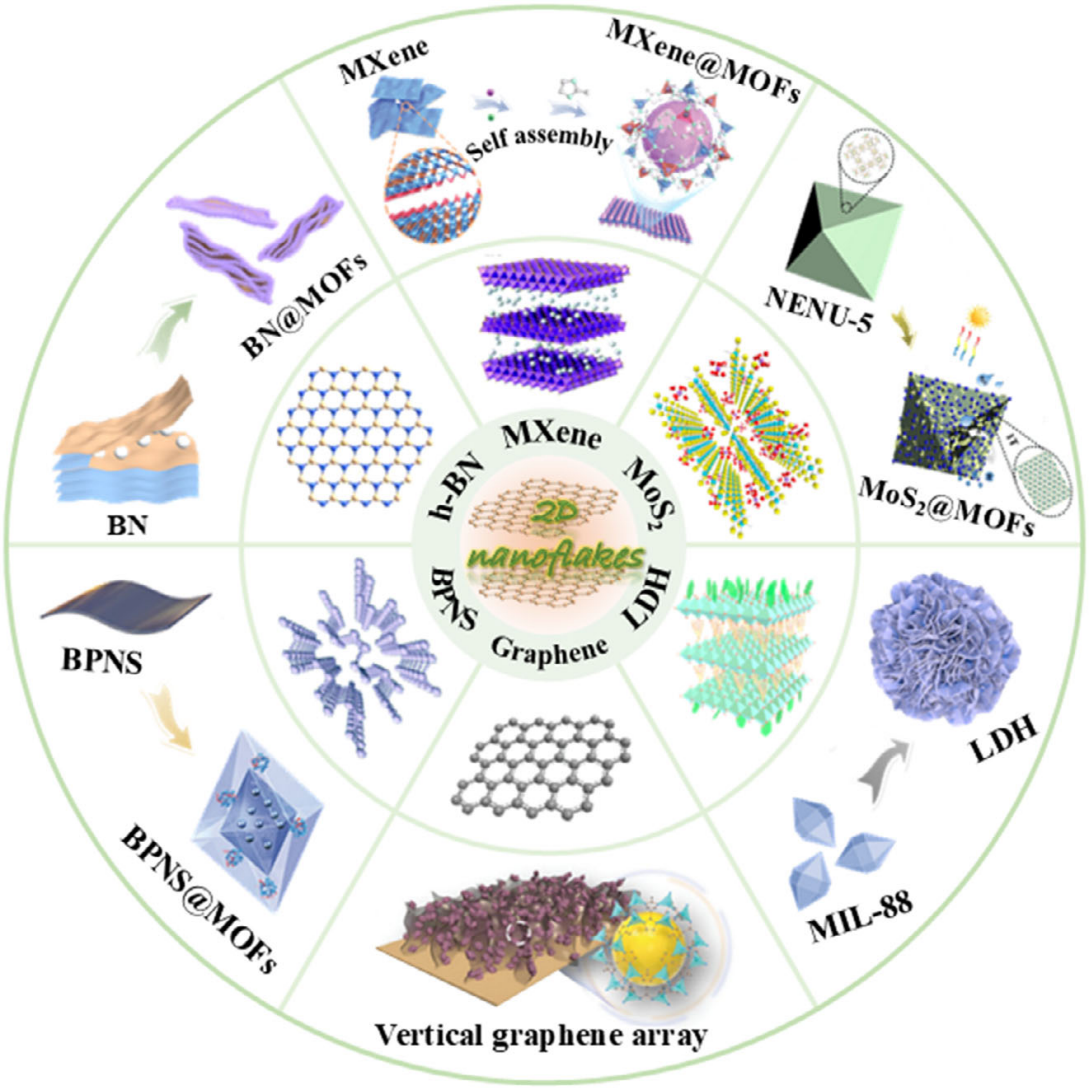
Schematic diagram of the composite mechanism of MOFs and typical 2D materials.^[^ ^98^, ^117^, ^118^, ^119^, ^120^, ^121^, ^122^, ^123^, ^124^ ^]^ (Reproduced (Adapted) with permission. Copyright 2024, John Wiley and Sons; Copyright 2021, John Wiley and Sons; Copyright 2023, Elsevier; Copyright 2019, John Wiley and Sons; Copyright 2022, Elsevier; Copyright 2020, Elsevier; Copyright 2020, Elsevier; Copyright 2020, John Wiley and Sons; Copyright 2020, American Chemical Society; Copyright 2017, Royal Society of Chemistry).

The integration strategy combining MOFs with 2D materials has emerged as a forefront research direction in the field of materials science, garnering considerable attention in recent years within the domains of gas separation, catalysis, electronic devices, and energy conversion. The objective of this integration is to amalgamate the respective advantages of MOFs and 2D materials, thereby generating novel composites with enhanced performance characteristics. On the one hand, the porous nature and large specific surface area of MOFs afford an abundance of active sites conducive to gas adsorption, molecular separation, and catalytic reactions. Conversely, the exceptional electrical, optical, and mechanical properties of 2D materials render them extensively utilized in electronic devices, optoelectronics, and energy storage applications. The primary integration strategies encompass the in situ growth of MOFs onto the surface of 2D materials, the utilization of 2D materials as structural units within MOFs, and the layer‐by‐layer assembly of MOFs and 2D materials. **Figure** **3** shows a schematic diagram of the widely used 2D material types and integration strategies with MOFs. These strategies not only realize the structural recombination of MOFs and 2D materials but also optimize the properties of materials through interfacial interaction. It provides new ideas for the design and development of new materials and is expected to achieve performance improvement and application expansion in many fields.

### 3.1 Physical Mixing Method

**Figure 4:**
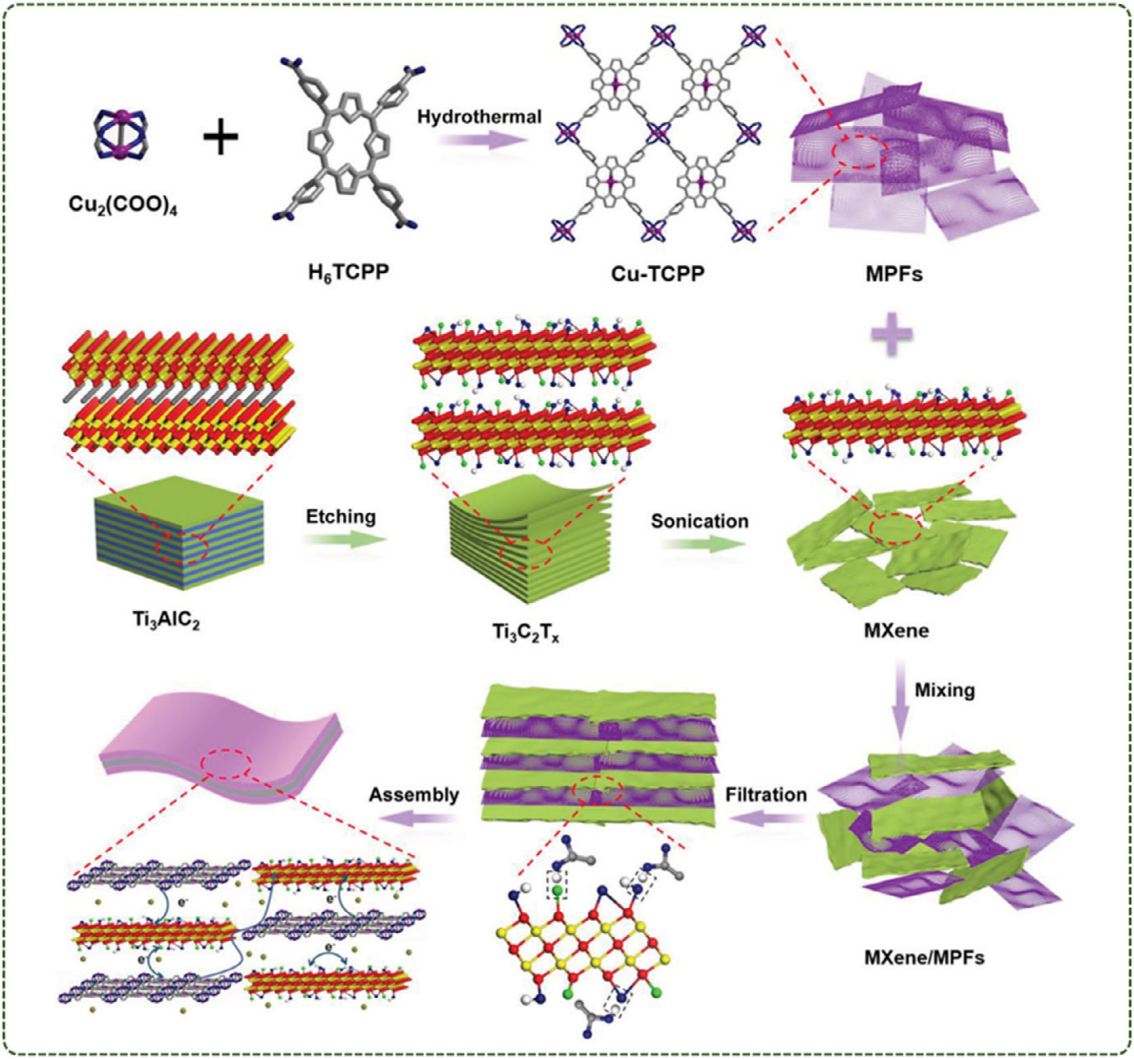
Schematic illustration of the synthesis and application of interlayer hydrogen‐bonded MXene/MPFs films through a vacuum filtration method (Reproduced (Adapted) with permission. Copyright 2019, John Wiley).

The physical mixing method is one of the simplest and most straightforward methods for compounding MOFs with 2D materials. In this method, MOFs are mixed with 2D materials in a polymer solution by simple mechanical stirring or ultrasonic dispersion, and then the composite materials are prepared by casting, coating, and other processes. For example, homogeneous mixing of MXene and MOFs and then processing the synthesized mixture under appropriate conditions can enable a simple combination of MXene and MOFs to form MXene@MOFs hybrids. Weak van der Waals interaction and hydrogen bond interaction are typical binding forces in the physical mixing method. Zhao et al.^[^ ^64^ ^]^ used the surface end of MXene to bind directly to hydrogen atoms in the carboxyl groups of MOFs nanosheets through hydrogen bonding. The self‐stacking of MXene and MOFs nanosheets is efficiently placed, and a 3D interconnected conductive network with a porous structure is provided to facilitate the rapid transport of ions and electrons (**Figure** **4**).

In addition, Sun et al.^[^ ^65^ ^]^ prepared novel 2D MOFs by solvothermal reaction one‐pot method and prepared BP nanosheets (BPNSs) by bulk crystal liquid‐phase exfoliation. It adheres to the surface of thionine‐doped 2D MOFs to form BPNSs/TH/Cu‐MOF hybrid materials. Zhang et al.^[^ ^66^ ^]^ prepared MIP‐202 and few‐layer MXene were mixed by ultrasonication and carbonized under argon protection to obtain a carbonized MIP‐202/MXene composite. Sun et al.^[^ ^67^ ^]^ prepared Mn‐doped Ni‐based MOFs with 3D‐layered flower‐like superstructure by solvothermal synthesis. Mn‐MOF and 2D BP nanosheets are then assembled together by hybrid ultrasound to form emerging 2D/3D nanocomposites. Although the physical mixing method is easy to operate, the main challenge is to ensure uniform dispersion and efficient interfacial interaction between MOFs and 2D materials in the polymer matrix. Therefore, in order to improve the properties of composites, it is often necessary to take auxiliary measures such as the use of surfactants, surface modification, or optimization of dispersion processes.

### 3.2 Chemical Bonding

Chemical bonding involves covalently bonding MOFs to 2D materials to form a tighter and more stable composite. This method can significantly improve the interfacial interaction between MOFs and 2D materials, thereby improving the overall properties of composites. Chemical bonding typically consists of two steps. First, active functional groups are introduced on the surface of MOFs or 2D materials through chemical methods (e.g., graft polymerization and cross‐linking reactions). These functional groups are then used to chemically react with polymers or other components to form chemical bonds.

**Figure 5:**
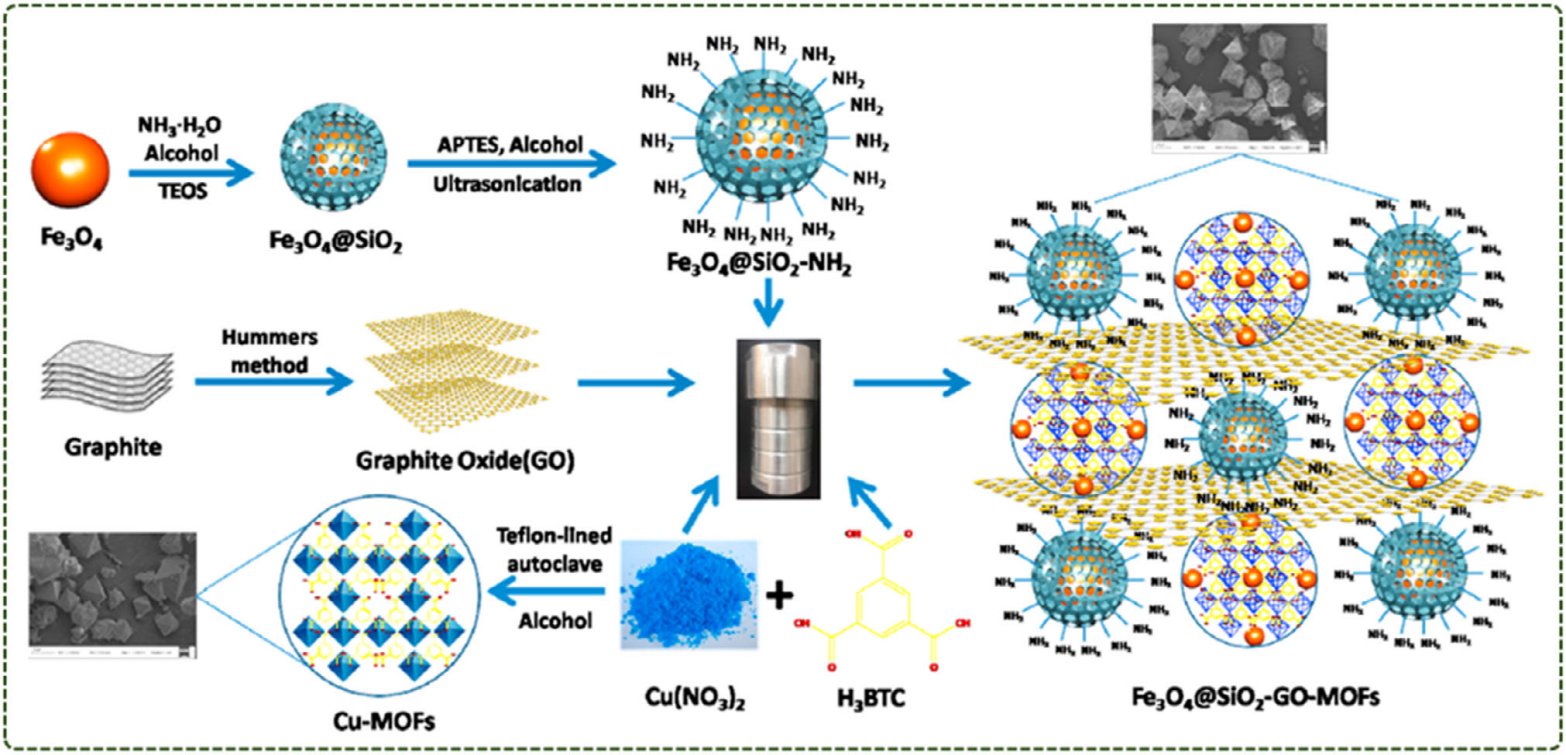
Magnetic Cu‐MOFs embedded within graphene oxide nanocomposites for enhanced preconcentration of benzenoid‐containing insecticides (Reproduced (Adapted) with permission. Copyright 2018, Elsevier).

Wang et al.^[^ ^68^ ^]^ covalently bonded GO nanosheets with silica‐coated Fe_3_O_4_ with a core–shell structure and synthesized a novel magnetic nanoparticle, and then modified the GO surface with Cu‐MOFs (**Figure** **5**). The nanocomposites combine the advantages of GO's large specific surface area with the magnetic properties of Fe_3_O_4_. This integration can be achieved by simply modifying the silica shell with an amine group on the GO nanosheet and then binding the amine to a carboxyl chemical bond. The silica shell of the composite material not only protects Fe_3_O_4_ particles from oxidation and agglomeration but also serves as a platform for the covalent bonding of Fe_3_O_4_ particles to GP nanosheets.

Shi et al.^[^ ^69^ ^]^ coordinated MXene with amine‐functionalized MOFs (UiO‐66‐NH_2_) through a carboxy‐assisted coordination pathway, resulting in tightly linked MOFs/carboxyl‐functionalized MXene heterostructures. This is the first time that MOFs heterostructures have formed chemical bonds by coordination bonds. Wang et al.^[^ ^70^ ^]^ established stable heterostructures using the electrostatic interaction between porphyrins and oxygen‐containing groups and the metal coordination of carboxyl groups in GO nanosheets. The use of GO‐like MOFs nanosheets facilitates the formation of nanofolded surfaces with vertical slits and in‐plane pores. Jayaaramulu et al.^[^ ^71^ ^]^ linked UiO‐66‐NH_2_ to carboxylic acid‐functionalized GO via amide bonds. The resulting nanohybrids have a large surface area.

The chemical bonding method not only enhances the interaction between MOFs and 2D materials but also improves the dispersion and stability of composites in the polymer matrix. For example, by linking GO nanosheets with MOFs through graft polymerization, composites with excellent mechanical properties and thermal stability can be prepared. In addition, chemical bonding can be used to manipulate the structure of composites to optimize their properties. For example, by changing the way MOFs are connected to 2D materials and the position of the connection points, the pore structure and surface properties of the composite materials can be manipulated to meet specific application needs.

### 3.3 Self‐Assembly Method

Self‐assembly is a method that uses intermolecular interaction forces (such as electrostatic interaction, hydrogen bonding, and *π−π* stacking) to spontaneously assemble MOFs and 2D materials into ordered structures. This method can not only realize the orderly arrangement of MOFs and 2D materials at the nanoscale but also adjust the structure and properties of the composites by adjusting the self‐assembly conditions. Xiao et al.^[^ ^72^ ^]^ proposed an effective interface engineering strategy to prepare MOFs nanocrystals tightly encapsulated by reduced GO (rGO) through strong chemical interactions. Through the synergistic coordination and electrostatic interaction between rGO and Co‐MOF, the Co‐MOF nano‐extraction with an average diameter of about 70 nm was evenly anchored on rGO. After further annealing, a hybrid monomer composed of Co‐MOF nanocrystals was obtained.

**Figure 6:**
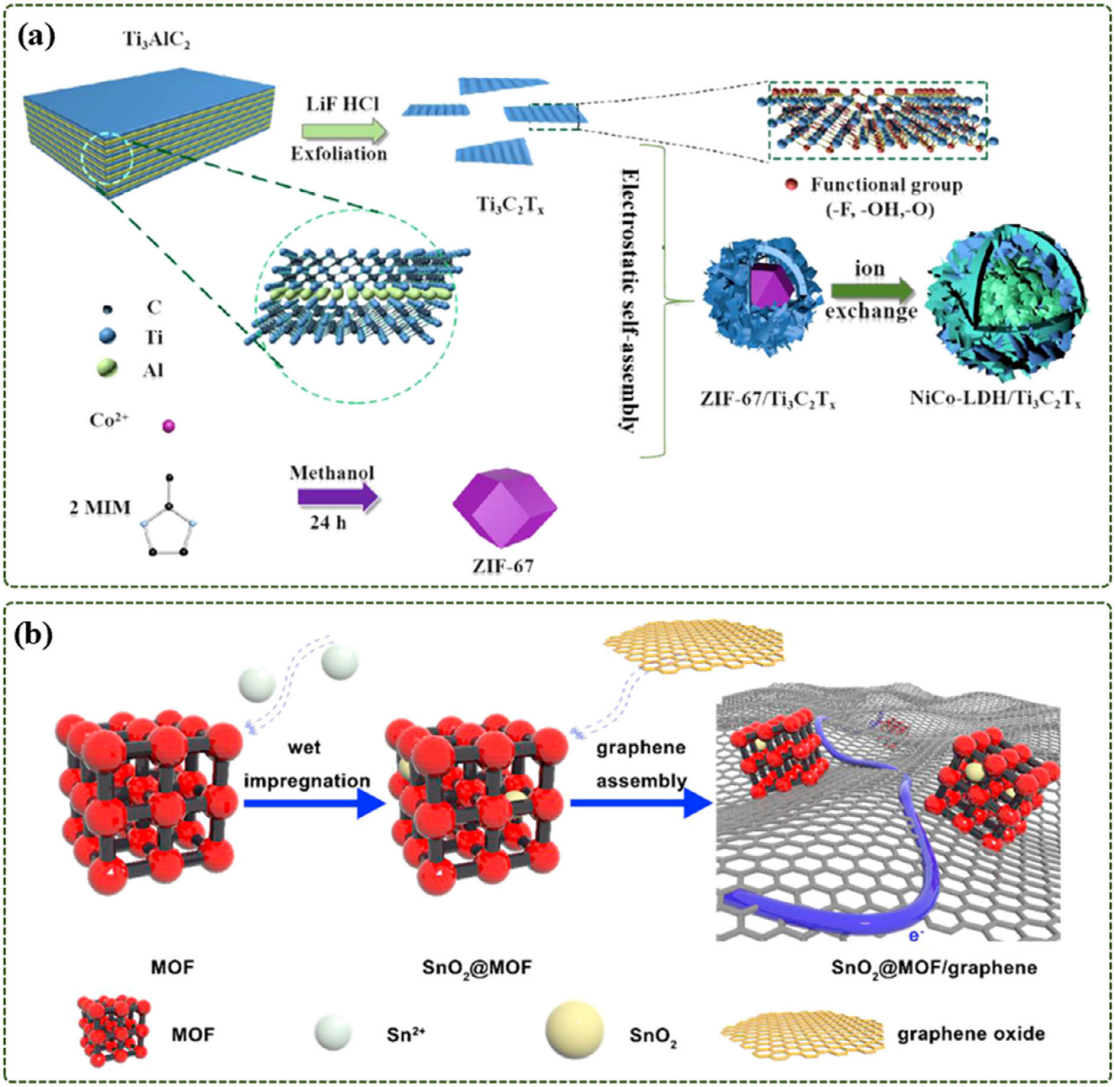
a) Schematic illustration of synthesis process of NiCo‐LDH/Ti_3_C_2_T_x_ (Reproduced with permission. Copyright 2021, Elsevier); b) preparation of SnO_2_@MOF and SnO_2_@MOF/graphene (The blue line in SnO_2_@MOF/graphene represents the electron transport path) (Reproduced (Adapted) with permission. Copyright 2020, Elsevier).

Wang et al.^[^ ^73^ ^]^ encapsulated the ZIF‐67 dodecahedron with MXene nanosheets through electrostatic self‐assembly to form a spherical ZIF‐67/MXene shape. The 3D floral NiCo‐LDH/MXene was then derivatized by a solvothermal ion exchange reaction (**Figure** **6**a). This hybrid material allows MXene to be evenly distributed and in close contact with NiCo‐LDH, which can effectively improve the conductivity of NiCo‐LDH. Chen et al.^[^ ^74^ ^]^ reacted with MXene by hydrogen bonding to generate Co‐TCPP(Fe)/MXene hybrids for NO sensing. Gao et al.^[^ ^75^ ^]^ proposed a simple strategy to optimize the assembly of SnO_2_ with MOFs and GO. The SnO_2_ nanoparticles were encapsulated in Al‐MOF at the optimal mass ratio, and then the resulting composite material was wrapped in GO, and finally the SnO_2_@MOF/GO composite material was obtained (Figure 6b).

The key to the self‐assembly method is to select the appropriate MOFs and 2D materials, as well as to regulate the various conditions (e.g., temperature, pH, and solvent) in the self‐assembly process. By optimizing these conditions, it is possible to achieve a uniform dispersion and orderly arrangement of MOFs and 2D materials in the polymer matrix, thereby improving the overall performance of the composite. For example, composites with excellent conductivity and mechanical properties can be fabricated by assembling positively charged MOFs with negatively charged GO nanosheets through electrostatic interactions. In addition, the self‐assembly method can also be used to prepare composite materials with special structures and functions. For example, by adjusting the temperature and solvent conditions in the self‐assembly process, MOFs/2D material composites with multilevel structures can be prepared, and these composites have potential application value in gas separation, energy conversion, and other fields.

### 3.4 In Situ Growth Method

In situ growth is a method for the in situ synthesis of MOFs and 2D materials in a polymer matrix. This method involves the addition of a precursor of MOFs and a growth substrate of the 2D material to the polymer matrix. The reaction is then carried out under specific conditions (e.g., temperature, pressure, and solvent) to grow and recombine the MOFs with the 2D material in the polymer matrix. The advantage of the in situ growth method is that it can achieve uniform dispersion and tight binding of MOFs to the 2D material in the polymer matrix, thereby improving the overall performance of the composite. In addition, the structure and properties of MOFs and 2D materials can be manipulated by adjusting the conditions during in situ growth to meet specific application needs. For example, the in situ growth method can be used to synthesize MOFs/GO composites with specific morphology and pore structure in polymer matrix, which can significantly improve the adsorption performance and catalytic activity of the composites.

**Figure 7:**
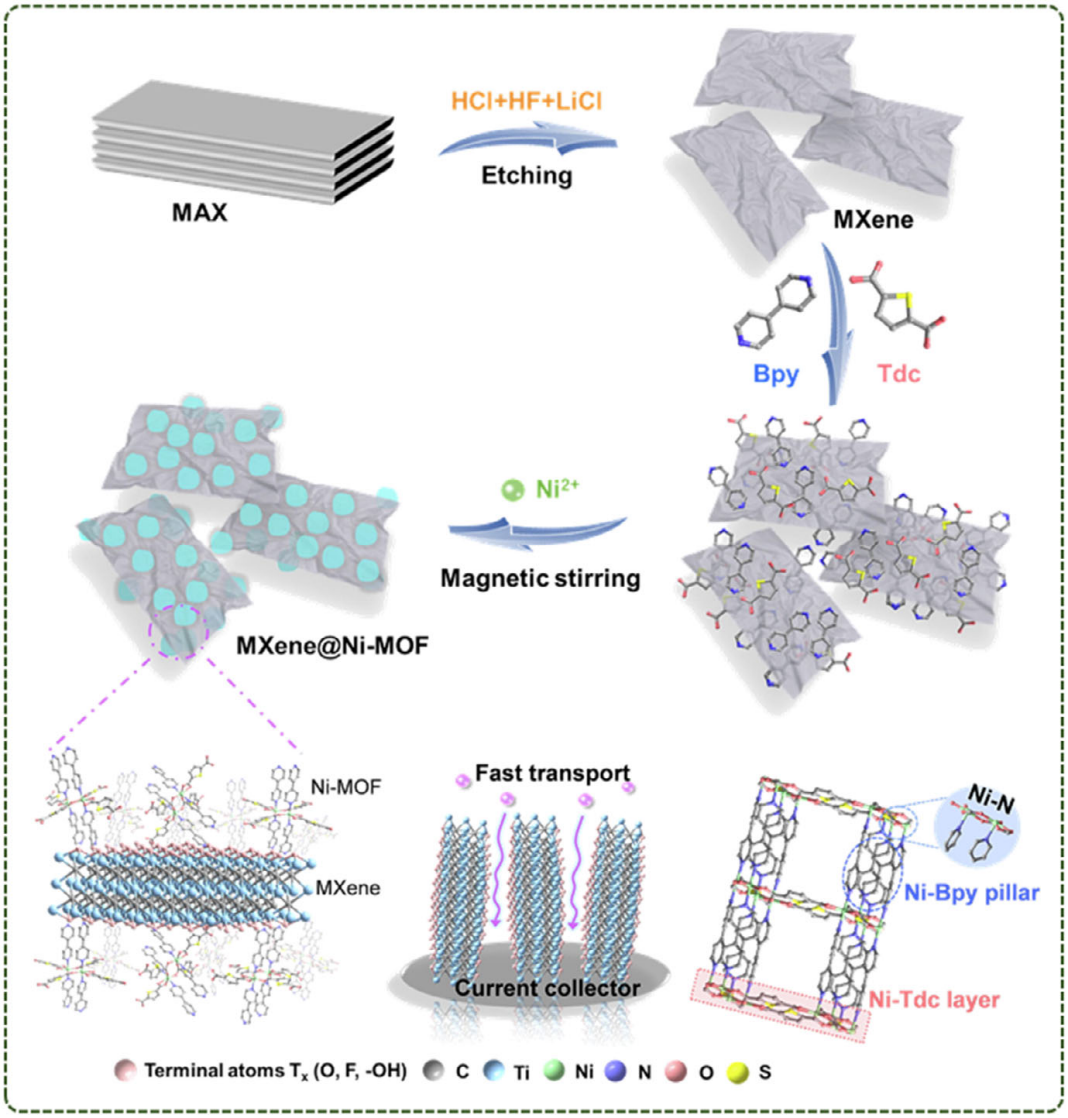
Schematic illustration of the preparation of MXene@Ni‐MOF, schematic diagram of the structure of the MXene@Ni‐MOF, schematic illustration of ion transport in MXene and pillar‐layered structure of Ni‐MOF (Reproduced (Adapted) with permission. Copyright 2022, Elsevier).

Zheng et al.^[^ ^76^ ^]^ used the interaction between the functional groups on the surface of MXene and the organic ligand of Ni‐MOF to inhibit the oxidation of MXene and increase its interlayer spacing. Then, Ni ions were introduced to grow Ni‐MOF on the MXene surface in situ to prepare MXene@Ni‐MOF hybrid materials. Fast charge transfer between Ni‐MOF and MXene was achieved and agglomeration of Ni‐MOF nanosheets was avoided (**Figure** **7**).

Zhang et al.^[^ ^77^ ^]^ used MXene nanosheets to provide abundant nucleation sites for the growth of MOFs and converted MOF nanospheres into layered porous nickel phosphate nanospheres in situ through “top‐down” etching. Finally, the porous nickel phosphate nanospheres were immobilized on MXene nanosheets, which solved the problem of low conductivity of nickel phosphate. Ge et al.^[^ ^78^ ^]^ synthesized BP/2D MOF(BP@MOF) heterojunctions by growing 2D MOF‐Fe/Co nanosheets in situ on the surface of BP nanosheets at room temperature. Fe^3+^ and Co^2+^ ions are adsorbed to the surface of BP by lone pair of electron coordination with BP, forming an intimate interface with strong P‐metal interactions. Then, the organic ligand was introduced to coordinate with the metal ion to realize the in situ growth of MOF‐Fe/Co nanosheets on the BP surface.

However, in situ growth methods also face some challenges. For example, the control of reaction conditions, the mutual interference between MOFs and 2D materials in the composite process, and the purification of composite materials. Therefore, the in‐situ growth process needs to be carefully designed and optimized in practical applications to ensure the performance and stability of the composites.

### 3.5 Performance Optimization of MOFs and 2D Materials Integrated Composites

#### 3.5.1 Structural Control

Structural manipulation is one of the important means to optimize the performance of MOFs and 2D‐integrated composites. By adjusting the structural parameters (such as pore size, shape, and surface properties) of MOFs and 2D materials, the properties of composite materials can be precisely controlled. For example, by adjusting the pore size and shape of MOFs, the adsorption performance and separation efficiency of composites can be optimized. By changing the surface properties and thickness of the 2D material, the conductivity and mechanical properties of the composite can be controlled.

In addition, the performance of MOFs/2D material composites with multilevel structures can be further improved by designing them. For example, by growing a layer of GO nanosheets on the surface of MOFs particles, composites with core–shell structures can be formed. This structure can not only improve the stability and dispersion of MOFs but also enhance their catalytic activity and selectivity.

#### 3.5.2 Functionalization

Functionalization is another effective method to optimize the performance of composite materials integrated with MOFs and 2D materials. By introducing specific functional groups or chemical segments on the surface of MOFs or 2D materials, composites can be imparted with new functional properties. For example, the catalytic activity and selectivity of MOFs can be improved by introducing functional groups such as amino or carboxyl groups on the surface. By introducing fluorine‐ or sulfur‐containing functional groups on the surface of GO nanosheets, their surface properties and chemical stability can be improved.

Functionalized modifications can not only improve the properties of composite materials but also expand their range of applications. For example, the application of MOFs/2D material composites to the field of sensors through functionalization can achieve high sensitivity and selectivity for the detection of specific gases or ions. By applying it to the field of energy conversion, the efficiency and stability of solar cells or fuel cells can be improved.

#### 3.5.3 Interface Interactions

Interfacial interaction is one of the key factors affecting the performance of MOFs and 2D integrated composites. By enhancing the interfacial interaction between MOFs and 2D materials, the overall performance and stability of composites can be improved. To achieve this, there are several strategies that can be employed to enhance interface interactions. A common approach is to use chemical bonding to link MOFs to 2D materials. By introducing specific functional groups or chemical segments, strong interaction forces such as covalent bonds or ionic bonds are formed between MOFs and 2D materials, so as to improve the mechanical properties and thermal stability of composites. Another approach is to use additives such as surfactants or polymers to improve the dispersion and compatibility of MOFs with 2D materials in the polymer matrix. These additives can interact with MOFs and 2D materials through weak interaction forces such as electrostatic interactions and hydrogen bonds, thereby enhancing the interfacial interaction forces between them.

In addition to the above methods, interfacial interactions can also be enhanced by optimizing the preparation process of composites. For example, by controlling conditions such as temperature, pressure, and reaction time during the compounding process, the dispersion state and interface structure of MOFs and 2D materials in the polymer matrix can be optimized, thereby improving the overall performance and stability of the composite.

## 4 The Integration of MOFs and 2D Materials in the Application of Polymer Materials

The central idea of the integration strategy of MOFs and 2D materials is based on overcoming the shortcomings of each component while establishing synergistic interactions that enhance the intrinsic properties. For example, MOFs are porous and have a large specific surface area, while 2D materials have excellent electrical, optical, and mechanical properties. Therefore, the hybrids of MOFs and 2D materials can not only improve conductivity and porosity but also enhance specific physicochemical properties with long‐term stability. Given the versatility of this 2D material@MOFs, they are widely used in a variety of applications. According to the literature survey, the most studied 2D materials are mainly GO and MXene. **Table** **1** summarizes the latest advances in the integration of MOFs, with potential applications in areas such as flame retardant, sensing, catalysis, and energy storage/conversion. This section focuses on the application of MOFs integrated with 2D materials in the field of flame retardant and sensing. The bibliometric method is used to visually analyze the application of MOFs integrated with GO and MXene in the field of flame retardant and sensing.

**Table 1:** A variety of 2D materials@MOFs and their design strategies for various applications.

No.	2D materials @MOFs	Design	MOFs	Applications		References
1	GO@Zn/CoMOF	In situ synthesis with mixing	Zn/Co MOF	EP flame retardant	Flame retardant	[125]
2	GO‐g‐PPA/ZIF‐8	co‐precipitation	ZIF‐8	PLA flame retardant		[90]
3	Zn/Co MOF@GO	In situ synthesis	Zn/Co MOF	EP flame retardant		[91]
4	La‐MOF@MXene	In situ synthesis	La‐MOF	TPU flame retardant		[126]
5	Co‐MOF@MXene	Self‐assembling	Co‐MOF	TPU flame retardant		[92]
6	Ni‐MOF@MXene	chemical bonding and physical adsorption	Ni‐MOFs	TPU flame retardant		[127]
7	CoFe‐MOF@MXene	Self‐assembling	CoFe‐MOF	renewable wood‐derived porous carbon flame retardant		[128]
8	Ti3C2Tx/ZIF‐8 MOFs	In situ synthesis	ZIF‐8	PU flame retardant		[129]
9	BP‐CoNi‐ML	In situ synthesis	CoNi‐ML	EP flame retardant		[97]
10	BP@MIL‐53	Self‐assembling; in situ synthesis	MIL‐53(Al)	PC flame retardant		[96]
11	Ce‐MOF@MoS2	In situ synthesis	Ce‐MOF	EP flame retardant		[99]
12	G@Cu‐MOF	In situ synthesis with mixing	Cu‐MOF	EP flame retardant		[130]
13	Gr/FeCu‐NZs/Gce	Simple mixing	Fe‐MOF	H_2_O_2_ Sensing	Sensing	[102]
14	DLS‐2D‐Co‐TCPP(Fe)/ANS–rGO	Chemical bonding	DLS‐2D‐Co‐TCPP(Fe)	NO sensing		[131]
15	GA@UiO‐66‐NH_2_	Chemical bonding	UiO‐66‐NH_2_	CO_2_ chemical resistance sensing		[101]
16	Cu‐BTC‐GO	In situ synthesis	Cu‐BTC	Ammonia sensing		[132]
17	G‐Ni/C	In situ synthesis	Ni‐MOF	Electrochemical sensors		[133]
18	Fe_2_O_3_/GO	Electrostatic self‐assembly	Fe‐MOF	Room temperature gas sensing		[134]
19	MXene@CuO	Mixing with MXene	Cu‐MOF	Ammonia sensing		[135]
20	MXene/ZIF‐67/CNTs	In situ synthesis with mixing	ZIF‐67	Electrochemical sensors in pharmacology		[136]
21	MXene/ZIF‐8	In situ synthesis	ZIF‐8	Hydrazine sensing		[137]
22	Cu‐MOF/MXene/GCE	Simple mixing	Cu‐MOF	H_2_O_2_ sensing		[138]
23	NiO/MXene	In situ synthesis	Ni‐MOF	H_2_O_2_ sensing		[139]
24	BPNSs/TH/Cu‐MOF	Simple mixing	Cu‐MOF	Electrochemical sensors		[65]
25	AgNCs/BPQDs/MOF	Simple mixing	Zn‐MOF	Enzyme catalyzed bio‐sensing		[140]
26	ZIF‐8/rGO	Self‐assembly	ZIF‐8	Electrode material in energy storage devices	Energy Storage	[141]
27	Gr‐CNT@Co	Simple mixing	ZIF‐67	Electrode material in energy storage devices		[142]
28	c‐MOF@GNF	In situ synthesis	c‐MOF	Supercapacitor electrode material		[143]
29	rGO@CoFe‐MOF	Utrasound‐assisted	CoFe‐MOF	Supercapacitor electrode material		[144]
30	CoCe‐MOF@rGO/BPMW‐300	In situ synthesis	CoCe‐MOF	Supercapacitor electrode material		[145]
31	NiCo MOF/VGN	In situ synthesis	NiCo MOF	Supercapacitor electrode material		[146]
32	N‐MXene	In situ nucleation and conversion	ZIF‐67	Separator for Li‐S batteries		[147]
33	CoZn‐Se@N‐MXene	In situ synthesis	CoZn‐MOF	Electrocatalyst for Lithium polysulfide conversion in Li‐S batteries		[118]
34	MXene/NiCo‐MOF	Simple mixing	NiCo‐MOF	Lithium‐ion batteries		[148]
35	Co‐Fe oxide/MXene	MXene derived MOF	Co‐Fe oxide	Supercapacitor electrode material		[149]
36	Ni‐MOF/MXene	In situ synthesis	Ni‐MOF	Supercapacitor electrode material		[150]
37	NC‐MXene	In situ synthesis	ZIF‐8	Electrode material in energy storage devices		[151]
38	NCNRs@NCNSs	In situ synthesis; ion exchange	NiCo‐MOF	Electrode material in energy storage devices		[152]
39	Co‐MOF@CoNiO_2_	In situ synthesis	Co‐MOF	Supercapacitor		[153]
40	NiCo‐LDH	In situ conversion in electrolyte	MOF	Supercapacitor electrode material		[154]
41	3D‐NCMOF@MS NC	Simple mixing	NiCo‐MOF	Supercapacitor electrode material		[155]
42	Co‐CNS/MXene	In situ synthesis	Co‐MOF	Solar absorption	Water treatment	[156]
43	MXene/UiO‐66‐NH_2_	In situ synthesis	UiO‐66‐NH_2_	Water harvesting		[157]
44	Cu‐TCPP/GO	In situ synthesis	Cu‐TCPP	Waste water treatment		[158]
45	Co‐MOF/GO	Simple mixing	Co‐MOF	Photocatalytic H_2_ activity	Catalysis	[159]
46	BiVO_4_@NH_2_‐UiO‐66	Simple mixing	NH_2_‐UiO‐66	Photocatalytic desulfurization		[160]
47	GO/Fe‐TCPP	Chemical bonding	Fe‐TCPP	Electrocatalysis		[161]
48	HKUST‐1/MXene	In situ synthesis	HKUST‐1	Catalytic application		[162]
49	MXene/TiO_2_/UiO‐66‐NH_2_	In situ synthesis	UiO‐66‐NH_2_	Photocatalytic H_2_ activity		[163]
50	NiCoS/MXene	In situ nucleation and conversion	ZIF‐67	Electrocatalysis		[164]

### 4.1 Application in the Field of Flame Retardant

Flame‐retardant polymer materials occupy a pivotal position in modern technology and industrial applications, and their importance cannot be ignored. With the rapid development of science and technology, polymer materials have been widely used in many fields, such as construction, transportation, electronic and electrical, and textiles, and have become one of the indispensable basic materials for modern society. However, the flammability of polymer materials has always been a key factor restricting their safe application. The R&D and application of flame‐retardant polymer materials have effectively improved the safety performance of materials under fire conditions. These materials slow down the spread of fires during combustion, reducing smoke releases and toxic gas production, buying valuable time for evacuation and fire rescue.^[^ ^79^, ^80^, ^81^ ^]^ In addition, the use of flame‐retardant polymer materials also significantly reduces the property damage caused by fire, which is of great significance for ensuring public safety. The importance of flame‐retardant polymer materials is not only reflected in their wide application value but also in their far‐reaching impact on public safety, scientific and technological progress and discipline development. In the future, with the continuous improvement of people's requirements for material safety performance, the research and application of flame‐retardant polymer materials will usher in a broader development prospect.

Due to their excellent catalytic carbonization ability, transition metals can increase the density of cross‐linked networks during polymer combustion. Therefore, it can form a continuous and strong char layer, which effectively blocks heat transfer and achieves the purpose of flame retardant and smoke suppression. MOFs are functionalized porous crystalline materials formed by the linkage of transition metal centers and organic ligands through coordination bonds. Among them, transition metals catalyze the formation of char layers, nitrogen oxides produced by the pyrolysis of organic ligands dilute combustible gases, and unique pore structure adsorbs volatile products.^[^ ^82^, ^83^ ^]^ Since 2017, it has been widely used as a flame retardant in polymer materials. However, when MOFs are used as fillers alone, the improvement effect of flame‐retardant performance of polymer composites is limited.^[^ ^84^, ^85^ ^]^ In addition, MOFs particles tend to agglomerate on the surface, affecting their dispersion properties in polymers, resulting in a decrease in the overall properties of polymers. In view of this, the dispersion performance of MOFs in the polymer matrix was improved by uniformly dispersing MOFs on the surface of 2D materials and combining them with nanocomposites, which was helpful to improve the flame‐retardant efficiency of nanocomposite flame retardants in polymer materials.

Despite the promising potential of MOFs as flame retardant materials to enhance the flame resistance of polymer materials, their flame‐retardant efficacy does not scale linearly with increased MOF content. While a moderate elevation in MOF concentration can indeed bolster the flame‐retardant properties of polymers, an excessive amount introduces a multitude of challenges. In addition, the uniformity of dispersion of MOFs nanoparticles in polymer matrices is also a challenge. Excessive addition can easily aggravate the agglomeration phenomenon of MOFs, which further affects the overall performance of the material. Second, too high an addition amount often leads to the deterioration of the overall mechanical properties of the material. This is because the interfacial interaction between MOFs nanoparticles and the polymer matrix may not be strong enough, resulting in interfacial debonding and destruction of the material when stressed. Finally, from an economic point of view, too high an added amount will undoubtedly increase the production cost of the material and limit its promotion in practical applications.

The introduction of 2D materials can solve the agglomeration phenomenon of MOFs nanoparticles in polymer matrix to a certain extent. However, too much MOFs will also cause them to accumulate on the surface of 2D materials, which is easy to fall off. In the process of processing with the polymer matrix, the phenomenon of secondary agglomeration is formed, which in turn affects the performance of the matrix. In summary, it is particularly important to reasonably regulate the content of MOFs to achieve a balance between flame retardant properties and other properties of materials. At the same time, the intrinsic relationship between MOFs content and the flame retardant properties of polymer materials is deeply revealed, which can provide scientific theoretical basis and practical guidance for the research and development of high‐performance flame‐retardant materials.

#### 4.1.1 Application of MOFs and GO Integration in Polymer Flame‐Retardant Materials

As a new nanomaterial with unique physical and chemical properties, GO has shown great application potential in the field of flame retardant. Its 2D lamellar structure can be layered layer upon layer during the combustion process to form a dense physical barrier that effectively blocks air and heat transfer, thereby significantly improving the flame‐retardant performance of the material. In addition, GO can also cross‐link and compound with resin and other coating components to form a denser protective film and further enhance the flame‐retardant effect. At high temperatures, the GO coating produces carbon dioxide and water when burned and forms a dense char layer. This char layer has a better barrier effect, which can effectively reduce the temperature of the surface of the material and delay the propagation of flames.

**Figure 8:**
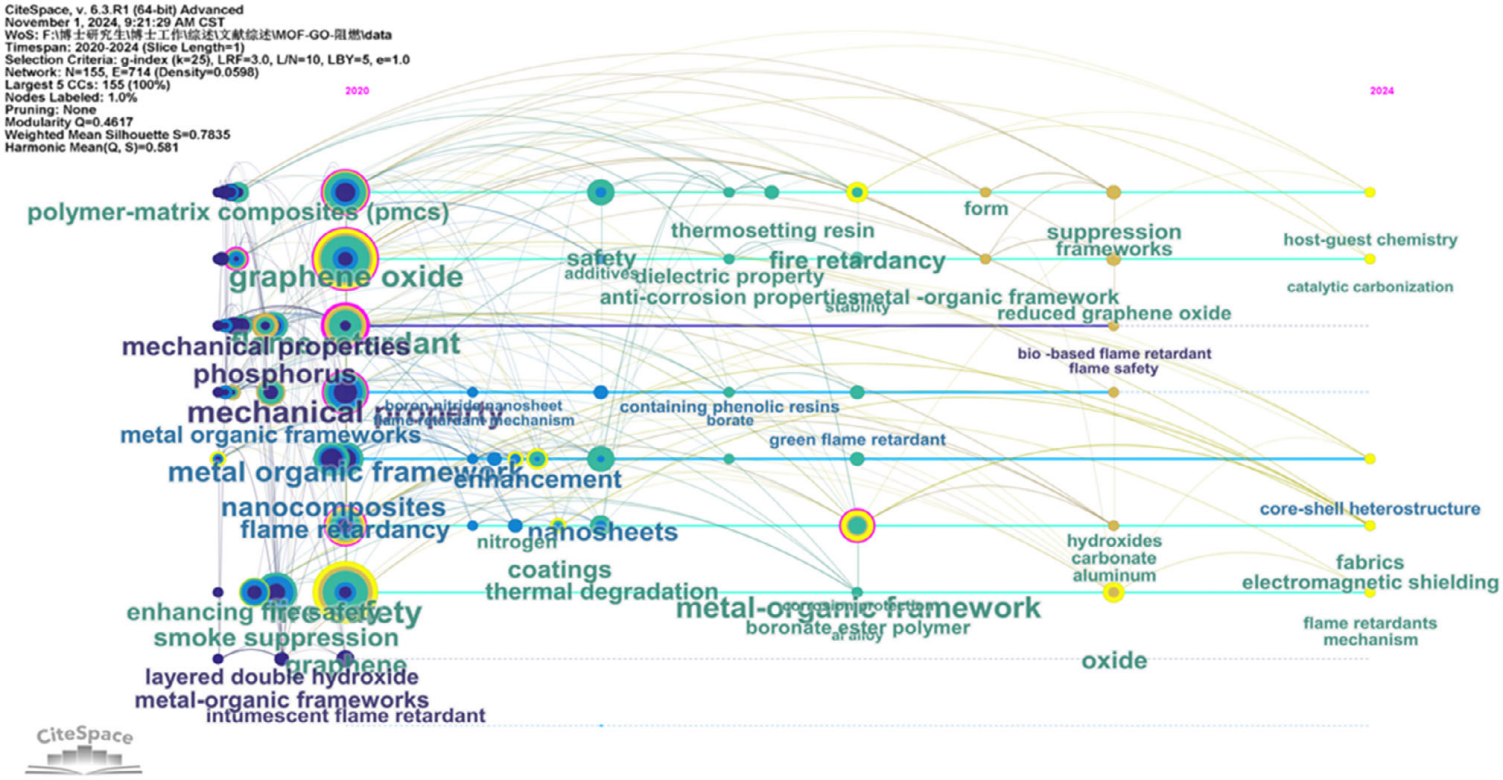
Frontier knowledge map of MOFs and GO synergistically improve the flame‐retardant properties of polymer materials in recent five years (2020–2024).

Bibliometrics provides a quantitative method for statistically and analyzing published literature in a specific field, facilitating access to detailed information such as authors, journals, keywords, and references.^[^ ^86^, ^87^, ^88^, ^89^ ^]^ In this article, the bibliometric method based on visual analysis software and the deep reading exploration method are used to analyze the integration of MOFs with 2D materials and their application in polymer materials in recent years. The chronological view can visualize the development process of MOFs and GO integration in flame‐retardant polymer materials (**Figure** **8**), so as to explore the focus and development direction of this field. Each colored circular node in the graph represents a leading hot spot, and the larger the node, the more frequent the keyword appears. In the past five years, research has mainly focused on flame‐retardant nanocomposites, MOFs and GO synergistically improve the flame‐retardant properties of polymer materials and improve the mechanical properties. Green bio‐based flame retardants and LDH have also received a lot of attention. Combined with the software tools that visualize the literature information, the development history of this field can be presented more clearly, which is convenient for us to select the core literature for review, analysis, and prospect in the next step.

**Figure 9:**
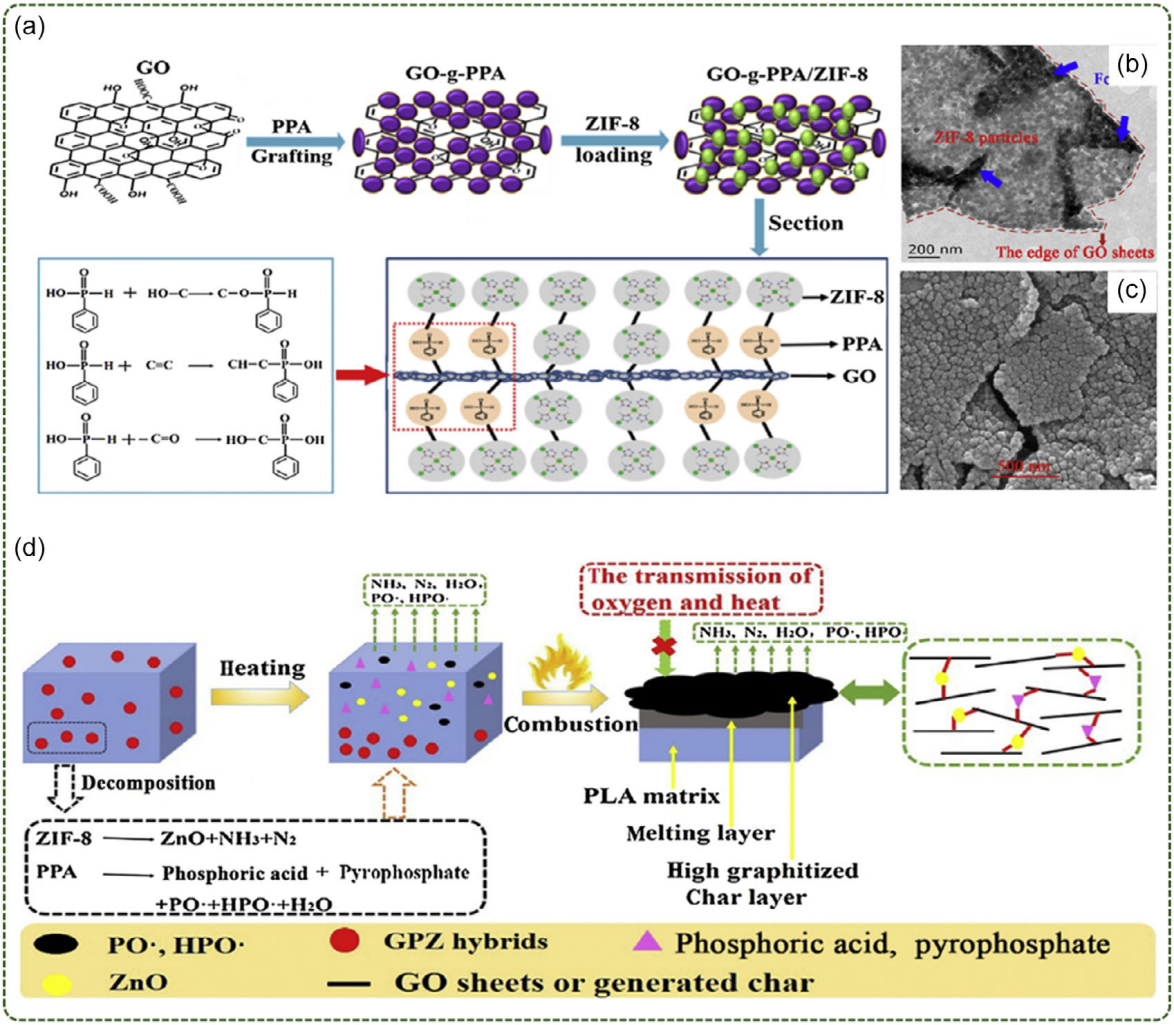
a) The synthesis of GPZ; b) TEM; c) SEM of GPZ; d) possible flame‐retardant mechanism of PLA nanocomposites (Reproduced (Adapted) with permission. Copyright 2020, Elsevier).

Wang et al.^[^ ^90^ ^]^ designed and synthesized a novel ternary hybrid nanosheet to improve the flame‐retardant properties of polylactic acid (PLA) using a simple two‐step method (**Figure** **9**a). They used “grafting” and co‐precipitation methods to construct a novel ternary hybrid flame retardant (GPZ) based on phenylphosphonic acid, ZIF‐8, and GO sheets. Figure 9b,c shows scanning electron micorscopy and transmission electron microscopy images of GPZ, respectively, confirming the successful synthesis of the lamellar hybrid material. Through this ternary hybridization, the prepared GPZ can obtain more flame‐retardant elements, including Zn, Co, P, and N elements, so as to achieve the purpose of high efficiency flame retardant. The experimental results show that the peak heat release rate (pHRR) of PLA composites is reduced by 39.5% with a GPZ of 2.0 wt% compared with pure PLA. The limiting oxygen index (LOI) value increased to 27.0%. At the same time, the total heat release (THR) of PLA composites is also significantly reduced. The composition and strength of the pyrolysis products of PLA composites were tested by thermogravimetric infrared (TG‐IR), and the results showed that the addition of GPZ inhibited the volatilization of pyrolysis products. In addition, the char residue after the cone size test also showed a higher degree of graphitization. This is related to the catalytic and cross‐linking of GO, ZIF‐8 and phenylphosphonic acid in the PLA matrix. Figure 9d shows the possible flame‐retardant mechanism of the system.

In addition, Wang et al.^[^ ^91^ ^]^ synthesized Zn and Co bimetallic organic backbone nanohybrid materials (MOF@GO) on GO with sandwich structure using an in situ growth method. By adjusting the ratio of Zn source and Co source, the size and distribution of Zn source and Co source were adjusted to make it grow evenly on the GO surface. When the ratio of Zn to Co was 6/4, the MOFs on the GO surface grew evenly. The nano‐hybrid materials show excellent thermal stability and can significantly improve the flame‐retardant properties of epoxy resins (EP). The 2.0 wt% MOF@GO material reduces the pHRR of the EP composites by 30%, and increases the LOI value from 23.4 to 29%. At the same time, MOF@GO nanocomposites significantly reduced the smoke toxicity of EP composites. The rate of CO release was reduced by 37% compared to pure EP. Compared to EP/MOF composites, EP/MOF@GO achieves better flame‐retardant properties. In this system, the physical barrier effect of layered GO and the excellent catalytic carbonization of transition metal‐based MOFs jointly achieved good flame‐retardant performance (Figure 9h). From this point of view, layered nano‐hybrid materials based on MOFs provide a new idea for reducing the fire hazard and mechanical properties of polymers.

#### 4.1.2 Application of MOFs and MXene Integration in Polymer Flame‐Retardant Materials

MXene is a GO‐like structure of 2D titanium carbide, which shows great application potential in the field of flame retardant. Its excellent thermoelectric properties and chemical stability make MXene an ideal flame‐retardant material. Studies have shown that MXene can not only maintain structural stability at high temperatures but it can also form strong interactions with other materials through the functional groups on its surface, thereby enhancing the overall flame‐retardant properties of the composite. In addition, the oxidation products of MXene, such as TiO_2_ nanoparticles, have a catalytic effect at high temperatures. This helps to create a more complete and denser layer of char, further inhibiting the spread of fire and the release of smoke. Integration with MOFs is also widely used in flame‐retardant polymer materials. However, the downside of MXene is that it is easy to restack. The functional modification of MXene can effectively solve this problem, so that MXene has good dispersion and compatibility in the polymer matrix.

**Figure 10:**
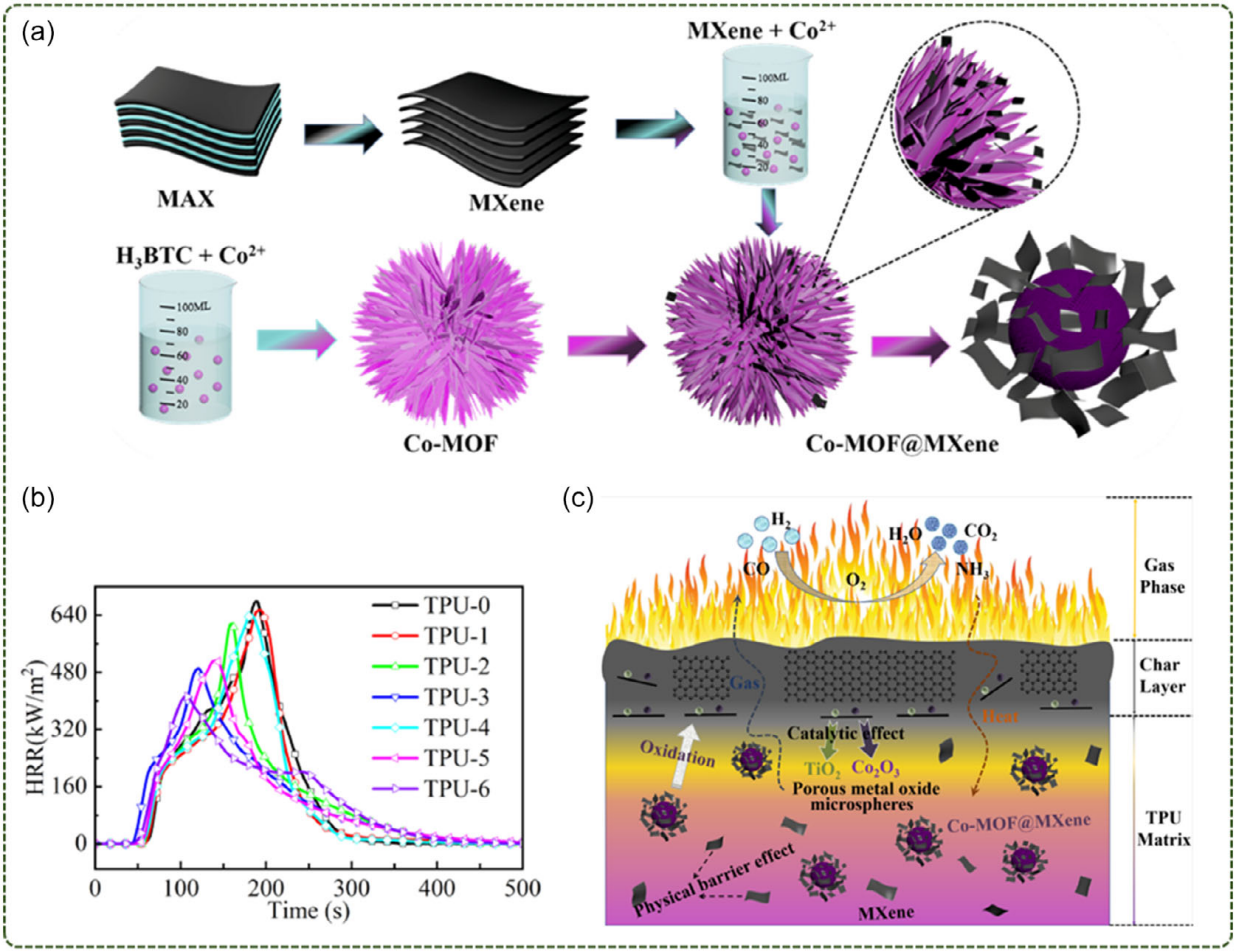
a) Schematic for synthetic routes of Co‐MOF@MXene hybrids; b) HRR curves of TPU and its composites; c) possible flame‐retardant mechanism of TPU/Co‐MOF@MXene composites (Reproduced (Adapted) with permission. Copyright 2022, Elsevier).

Shi et al.^[^ ^92^ ^]^ assembled MOFs with MXene nanosheets to form Co‐MOF@MXene based on the interaction between the end groups on MXene and the unsaturated sites of metal ions on MOFs (**Figure** **10**a). It is used to improve the thermal and flame‐retardant properties of TPU. The experimental results show that the incorporation of Co‐MOF and Co‐MOF@MXene leads to the early decomposition of TPU at low temperature, but the char residue of the composites is improved at high temperature. The char residue of the composites with 2 wt% Co‐MOF was 7.8% at 700 °C. However, the char residue of the composites with 2 wt% Co‐MOF@MXene reached 10% at 700 °C, which was greatly improved. The results of cone measurement show that the pHRR of TPU/Co‐MOF@MXene composites decreases more than that of TPU/Co‐MOF composites under loads of 0.5, 1, and 2 wt%. For example, composites containing 2 wt% Co‐MOF had a 28.3% reduction in pHRR values, while composites containing 2 wt% Co‐MOF@MXene had a 40.0% reduction in pHRR values (Figure 10b). The efficiency of Co‐MOF@MXene hybrids in reducing pHRR value was high, indicating that the catalytic carbonization of Co and Ti elements and the barrier effect of MXene could improve the fire performance of TPU matrix (Figure 10c). However, the long ultrasonic process required to prepare MXene nanosheets has the drawbacks of being time‐consuming and energy intensive.

**Figure 11:**
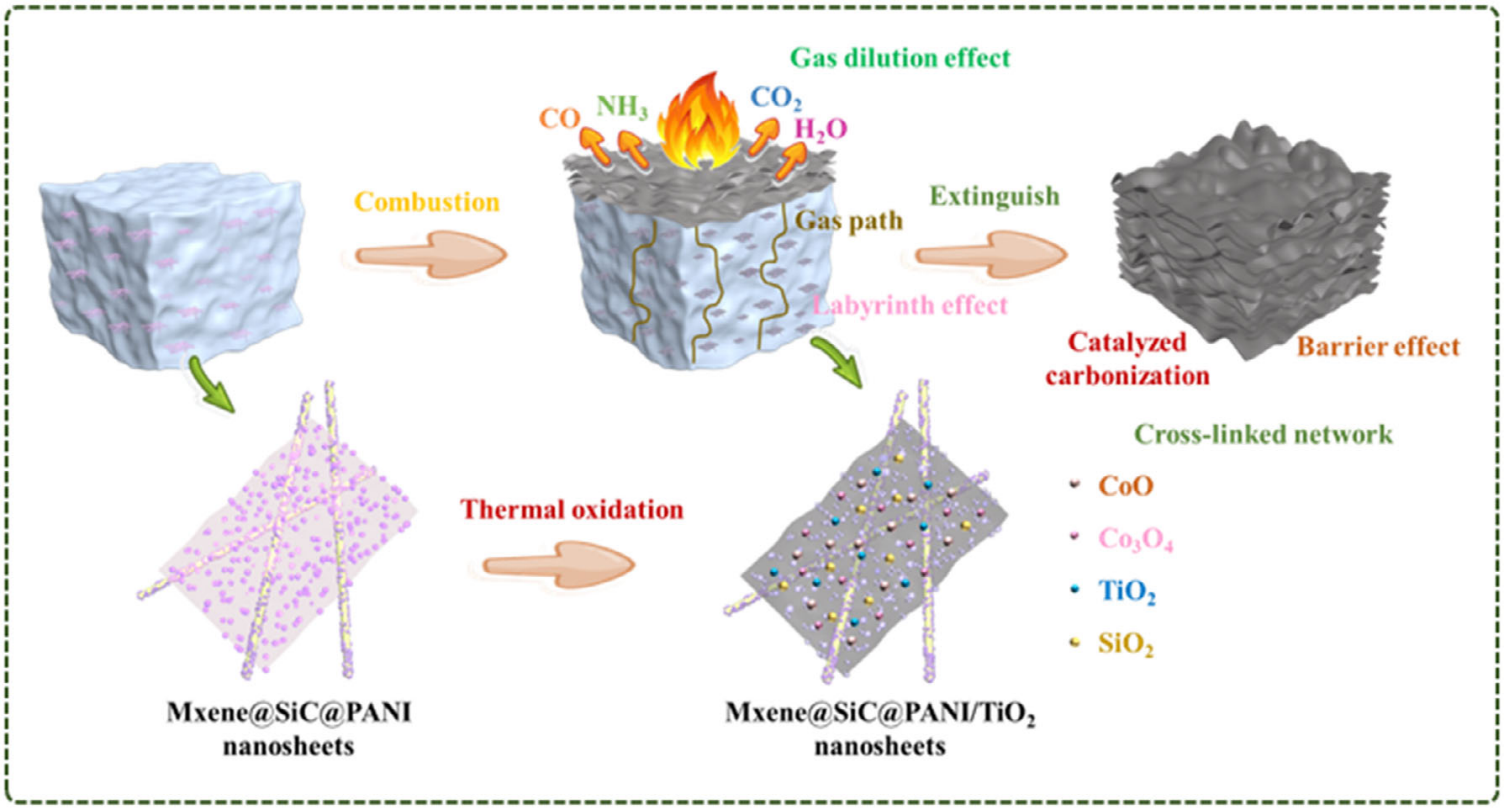
Schematic illustration of the flame retardant and smoke suppression mechanism (Reproduced (Adapted) with permission. Copyright 2024, John Wiley and Sons).

Bi et al.^[^ ^93^ ^]^ combined MXene with ZIF‐67 and introduced one‐dimensional silicon carbide (SiC) into the system, which easily realized the peeling modification of MXene. According to the previous work, polyaniline (PANI) was grown in situ on the surface of ZIF‐67, and finally a 2D hybrid material (MXene@SiC@PANI) with few or even single layers was obtained. It is also used to improve the flame retardant and smoke suppression properties of TPU. The TPU composite material increased from 33.4 to 59.8 °C in 10 s, showing good thermal conductivity. At the same time, TPU composites have a good effect on reducing the risk of fire. Compared with pure TPU, the pHRR and THR of TPU composites decreased by 71.4 and 34.6%, respectively. The results of TG‐IR showed that the hybrid material could significantly reduce the concentration of pyrolysis products. **Figure** **11** shows the possible flame‐retardant mechanism of the system. Firstly, the “labyrinth effect” produced by the 2D‐layered structure effectively prolongs the transport path of pyrolysis gas. Non‐combustible gases such as NH_3_ produced during the combustion process also play a role in diluting combustible gases. In addition, in the condensed phase, the catalytic carbonization of transition metal elements and the physical barrier of 2D materials provide a strong and dense char layer for the TPU matrix. As a result, the excellent flame‐retardant properties of TPU were obtained.

#### 4.1.3 Application of LDH in Polymer Flame‐Retardant Materials

LDH is a class of anionic compounds with broad application prospects, which shows great potential in the field of flame retardant. The flame‐retardant effect of LDH is mainly due to its unique layered structure and thermal stability. LDH decomposes when heated to a certain temperature, including steps such as interdelamination water, hydroxyl dehydration, and new phase generation. This process absorbs a large amount of heat, which helps to reduce the combustion temperature of the material. At the same time, the interlayer anions of LDH are interchangeable, and molecular design can be realized by introducing functional ions, thereby further enhancing its flame‐retardant properties. In recent years, many studies have shown that the combination of LDH and polymer materials after modification can significantly improve the flame‐retardant performance of the material. For example, through organic intercalation modification, LDH can be dispersed in the polymer matrix more effectively, improving the thermal stability and charring rate of the material, and reducing the combustion rate. In addition, the synergistic effect of LDH and other flame retardants also shows excellent flame‐retardant effect, which provides a new idea for the development of efficient and environmentally friendly flame‐retardant materials. Therefore, it is of great significance to study the flame‐retardant potential of LDH to promote the development of flame‐retardant materials.

Song et al.^[^ ^94^ ^]^ developed a unique multi‐yolk shell structure using ZIF‐67 and urea as templates and etchants using a simple one‐pot method. Its hybrid material consists of two cobalt‐based isomers, namely a Co‐LDH‐assembled nanocage and an egg yolk composed of cobalt‐alkaline carbonate. At the same time, the phosphorus‐based flame retardant triphenyl phosphate was introduced into the inner cavity and channel, and finally the m‐CBC@LDH nano hybrid flame retardant was obtained. PUA composites with 5 wt% m‐CBC‐P@LDH increased the LOI value to 22.6%, and decreased pHRR and THR by 41.7 and 20.6%, respectively. m‐CBC@LDH can firmly anchor TPP molecules in their channels and cavities. And with the collapse of the m‐CBC@LDH skeleton at high temperature, the anchored TPP will be released rapidly in a short time, so as to achieve synergistic flame retardant through a variety of flame‐retardant mechanisms.

**Figure 12:**
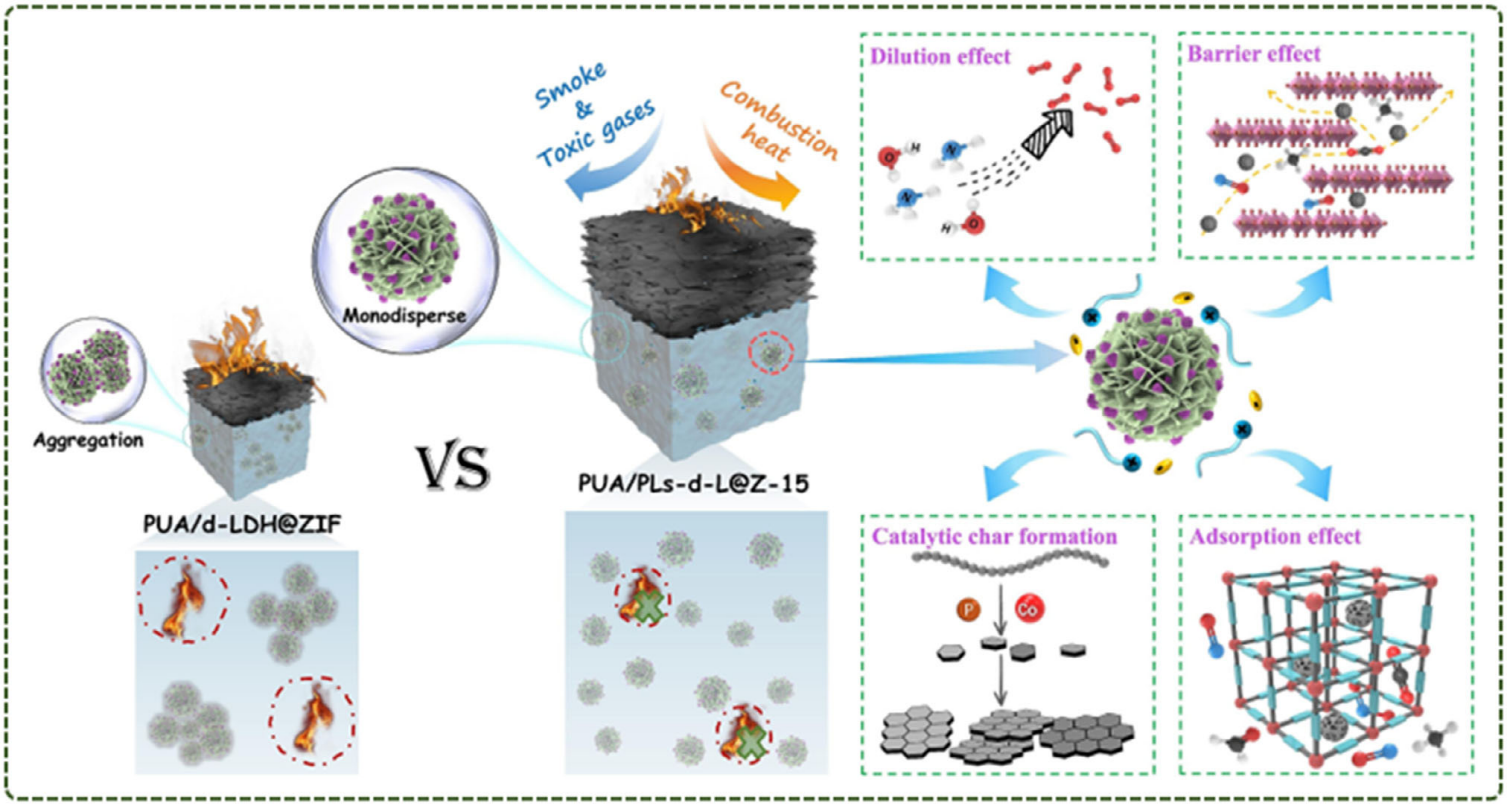
Schematic of the flame‐retardant mechanism for the PUA/PLs‐d‐L@Z composites (Reproduced (Adapted) with permission. Copyright 2024, Elsevier).

Further, they also constructed the defect‐Co‐LDH@ZIF‐67 (d‐LDH@ZIF) heterostructure in situ using Co‐LDH as a sacrificial template and vector.^[^ ^95^ ^]^ The lamination defects and low specific surface area defects of Co‐LDH were effectively improved. At the same time, due to the large number of cobalt/oxygen vacancies and lattice defects in the heterostructure, D‐LDH@ZIF is given higher intrinsic activity. It provides a promotion effect for the catalytic performance of hybrid materials. The flame‐retardant test results show that the D‐LDH@ZIF modified polyurea composites show good heat and smoke suppression properties. Among them, the PUA/PLs‐d‐L@Z‐15 specimen can self‐extinguish within 2 s after two ignitions, and the resulting molten droplets can take away the flame without igniting the absorbent cotton, reaching the V‐0 rating of UL‐94. The pHRR of PUA composites decreased by 40.4%, and the THR value also decreased to 65.5 MJ m^−2^. The possible flame‐retardant mechanism is shown in **Figure** **12**. At the beginning of combustion, D‐LDH@ZIF endothermic dehydration inhibits the temperature rise. The water vapor produced in this process acts as a dilution of the combustible gas and oxygen concentrations. In the later stages of combustion, d‐LDH@ZIF decomposes into cobalt oxides, catalyzing the cross‐linking of polymers into char. At the same time, the in situ grown ZIF‐67 expands the layer spacing between Co‐LDH nanosheets, thereby improving its catalytic and shielding properties. In summary, d‐LDH@ZIF gives the PUA matrix excellent flame‐retardant properties through a variety of flame‐retardant pathways.

#### 4.1.4 Application of MOFs and Other Two‐Dimensional Materials Integrated in Polymer Flame‐Retardant Materials

As a new type of 2D‐layered inorganic semiconductor material, BP has attracted extensive attention in the field of flame retardant in recent years. The results show that BP has a honeycomb‐like folded lamellar structure, which shows potential application prospects in optoelectronics, catalysis, energy storage, biomedicine, and other fields. In particular, BP and its derivatives generally have good flame‐retardant properties and can usually be combined with nitrogen, silicon, sulfur, and boron to form synergistic flame‐retardant materials. By stripping it into nanosheet layers, black phosphorene (BPNS) benefits from its 2D topography characteristics, inherent high strength, and outstanding thermal properties. It has the potential to be used as a nano‐additive to enhance the mechanical properties, thermal stability, and flame‐retardant properties of polymer materials. However, BPNS has poor stability in air and water environments and is prone to oxidation leading to performance degradation, which limits its effectiveness in practical applications. Therefore, how to improve the air stability of BP nanosheets through surface functionalization and other methods, while maintaining their excellent flame‐retardant properties, is the focus of current research.

**Figure 13:**
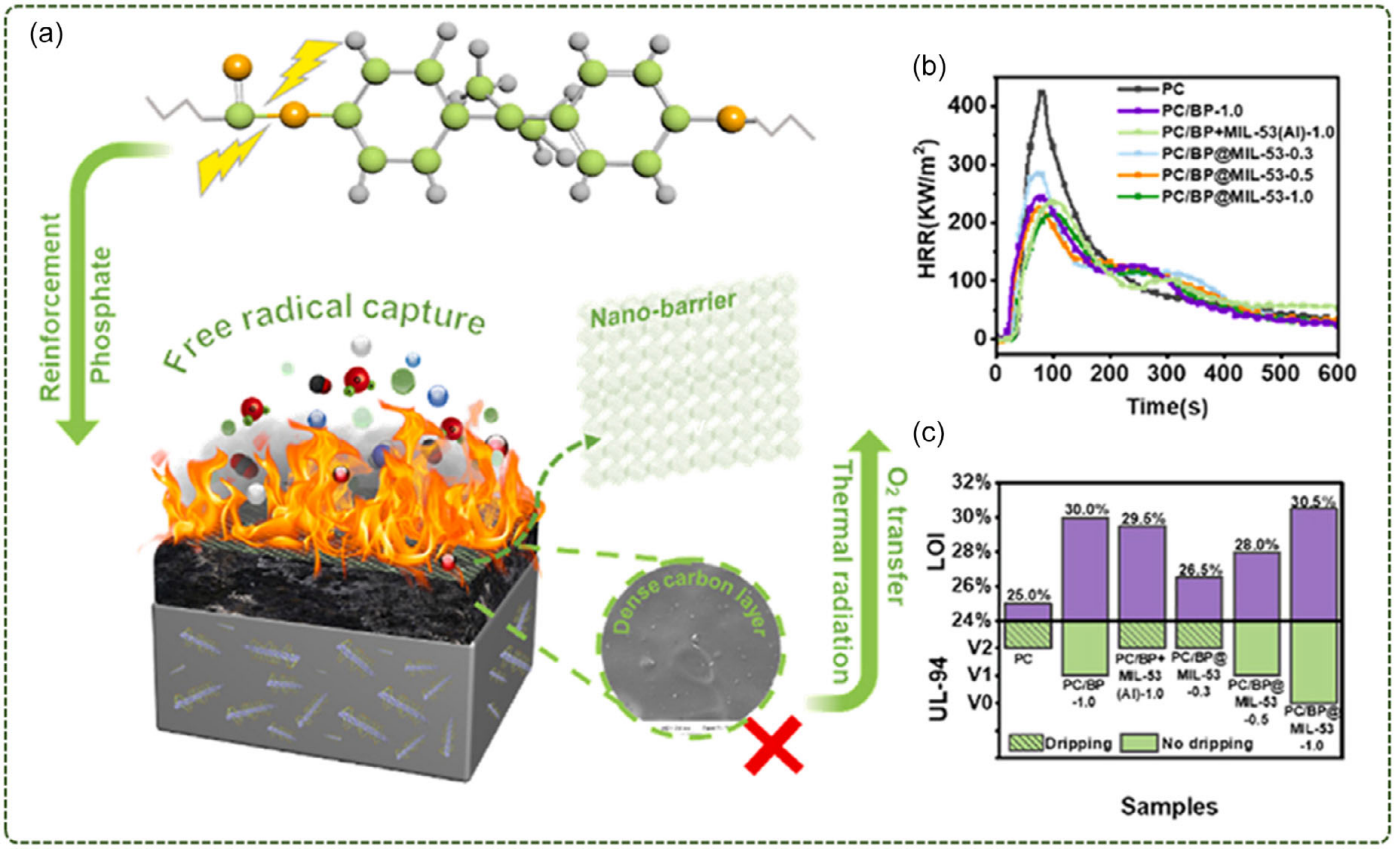
a) Schematic diagram of the mechanism of BP@MIL‐53 enhancing the fire safety and thermal stability of PC and its nanocomposites, b) HRR curves, c) LOI value and UL‐94 results of PC and its composites (Reproduced (Adapted) with permission. Copyright 2022, Elsevier).

Qian et al.^[^ ^96^ ^]^ used MIL‐53(Al) as a functionalized organic coating to modify the BP surface and prepared a BP@MIL‐53 hybrid material by hydrothermal method (**Figure** **13**a). The Al ions in MIL‐53 interacted with the lone pairs of electrons on the BP surface, and the organic ligand was further introduced by the in ‐situ growth method to make MIL‐53 stable on the BP surface. The obtained BP@MIL‐53 hybrid material is used to enhance the thermal stability and flame retardancy of polycarbonate‐based polymers. The test results showed that the pHRR of PC composites was significantly reduced when the addition amount was 1.0 wt%, which was 49.4% lower than that of pure PC. It is worth noting that the pHRR of PC/BP@MIL‐53‐1.0 composites with the same amount of filler are lower than that of PC/BP‐1.0. It was confirmed that the flame‐retardant efficiency of BP@MIL‐53 was better than that of BP with the same addition amount and higher phosphorus content. The HRR curve shown in Figure 13b shows a significant delay in the time to peak heat release for PC composites. And the PC composite achieves a UL‐94 V‐0 rating, which is very advantageous for real fire situations (Figure 13c).

Qiu et al.^[^ ^97^ ^]^ also proposed that the combination of MOFs with lone pairs on the surface of BP can reduce their surface electron density. LDH was generated in situ on MOFs on the BP surface, and a protective coating with high porosity and large specific surface area was created using a sacrificial template, called the BP‐CoNi‐ML nanolayer. Compared with pure EP, the LOI value of EP composites was significantly improved to 32.0, reaching a V‐0 rating in the UL‐94 test. The HRR curve shows that the pHRR and THR value decreases significantly as the BP‐CoNi‐ML loading increases. The BP‐CoNi‐ML/EP2.0 sample demonstrated a 58.8% decrease in pHRR and a 42.8% reduction in THR.

**Figure 14:**
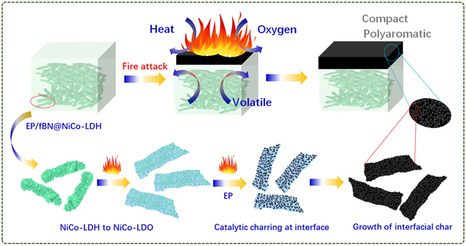
Multiscale fire‐retardant mechanism to link the structural basis of EP/fBN@NiCo‐LDH and fire performance (Reproduced (Adapted) with permission. Copyright 2023, Elsevier).

BN has an extremely high hardness and melting point at high temperatures, which can effectively prevent the spread of fire. When BN materials encounter an ignition source, a series of chemical reactions and physical changes occur that limit the spread and extension of the fire. In addition, BN nanosheets (BNNS) can act as a GO‐like physical barrier when polymers are burned, and are used as flame retardants. The flame‐retardant properties of BN can be further improved by means of surface functionalization. Zhang et al.^[^ ^98^ ^]^ used BN to enhance the flame retardant properties of EP without sacrificing mechanical properties. First, the bio‐based tannic acid (TA)‐assisted strategy was used to synchronously exfoliate and activate the BN, followed by osteoarticular inspired surface engineering with interlocking structures. A spatially adjustable stacking of layered NiCo‐LDH was introduced on the surface of activated BN using MOFs‐derived etching conversion mode. A hybrid flame retardant (fBN@NiCo‐LDH) of 6 wt% gave EP a UL‐94V‐1 rating and reduced pHRR generation by 30.0%. A flame‐retardant mechanism diagram is shown in **Figure** **14**. A compact and continuous carbonization structure with a higher degree of polyaromatic hydrocarbons is formed through interfacial carbonization, so better flame‐retardant properties are obtained.

In addition, MoS_2_, as an important inorganic compound, shows great potential in the field of flame retardant due to its unique structure and properties. As a commonly used flame retardant, MoS_2_ is not only stable at high temperatures and chemically stable but also favored for its low toxicity. At high temperatures, MoS_2_ is able to oxidize and decompose, releasing sulfides and gases, effectively inhibiting the combustion reaction. Thus, significantly improving the flame‐retardant properties of polymer materials such as plastics, rubber, polyester, and epoxy resins. In addition, MoS_2_ can be used in coatings and textiles to enhance its flame resistance.

**Figure 15:**
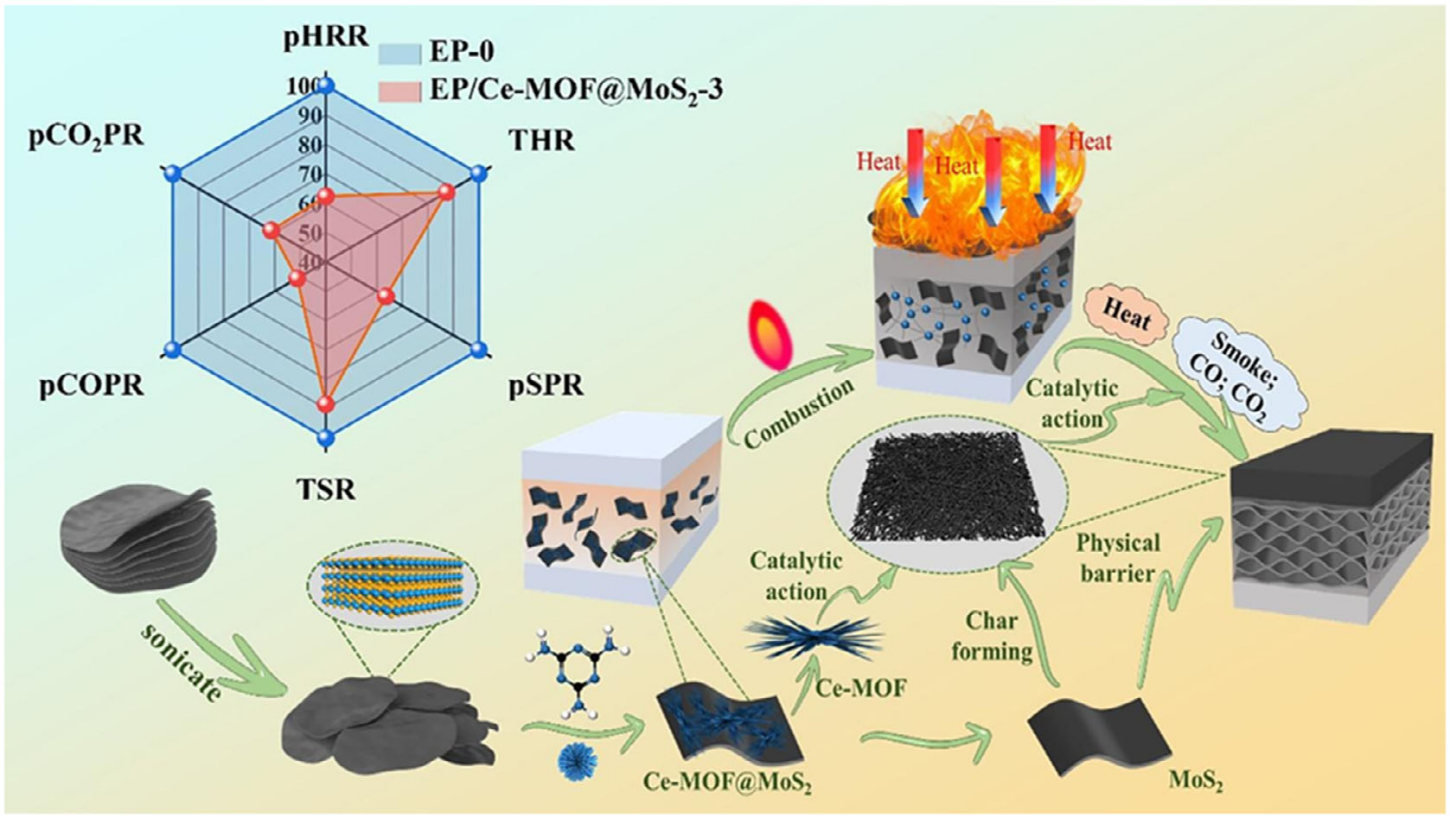
Graphical abstract of Ce‐MOF@MoS_2_ hybrid (Reproduced (Adapted) with permission. Copyright 2025, Elsevier).

Yu et al.^[^ ^99^ ^]^ prepared needle‐like Ce‐MOF crystals on MoS_2_ nanosheets using in situ growth techniques. This hybrid structure significantly improves the thermal stability and mechanical properties of EP. Compared with pure EP, the pHRR and THR of EP/Ce‐MOF@MoS_2_‐3 were reduced by 38 and 12.64%, respectively. In addition, Ce‐MOF@MoS_2_ inhibits smog and reduces the emission of toxic substances. When the loading was 3%, the CO and CO_2_ yields of EP nanocomposites were reduced by 48.8 and 38.7%, respectively. According to the analysis of the condensed phase and gas phase products of EP composites, it is found that the layered structure of Ce‐MOF@MoS_2_ promotes the formation of dense char layer. It effectively blocks the release of heat and oxygen and protects the polymer matrix. At the same time, the Ce‐MOF@MoS_2_ hybrid materials prepared by in situ growth technology improved the dispersion in the EP matrix and gave full play to their synergistic flame‐retardant advantages. The metal‐catalytic effect of Ce‐MOF and MoS_2_ effectively absorbs the smoke and toxic gases generated during the combustion of EP composites and finally achieves a better flame‐retardant effect (**Figure** **15**).

In order to clarify the importance of the integration of MOFs and 2D materials, the effects of MOFs, 2D materials and hybrids formed by them on the peak heat release rate of polymer matrices are summarized in **Table** **2**. Compared with MOFs and 2D materials alone, the flame‐retardant performance of hybrids on polymer matrices is more significantly improved.

**Table 2:** Effect of different MOFs, 2D materials, and their hybrids on the peak heat release rate of polymers.

No.	Matrix	Content	Flame retardancy (%)			References
			MOF (Alone)	2D materials [%]	MOF with 2D materials [%]	
1	EP	2	−10.9	−1.0	−29.4	[91]
2	TPU	2 wt%	−28.3	–	−40.0	[92]
3	EP	1.86 wt%	−16.4	−30.2	−37.7	[99]
4	TPU	2%	−15.0	–	−19.5	[127]
5	PLA	2 wt%	−18.1	–	−23.5	[165]
6	TPU	2 wt%	−35.3	–	−42.9	[166]
7	EP	2%	−24.2	–	−28.8	[167]
8	EP	6 wt%	−41.7	–	−54.9	[130]
9	EP	2 wt%	–	−44.1	−42.8	[97]
10	PC	1 wt%	–	−37.8	−49.3	[96]
11	TPU	5 wt%	–	−45.4	−71.4	[93]

### 4.2 Application in the Field of Sensing

As a key pillar of modern information technology architecture, sensing technology not only promotes the rapid development of the information society but also profoundly affects the innovation process in many high‐tech fields. In this technology system, the introduction and application of functional polymer composite materials are gradually becoming a research hotspot, and their potential in the field of sensing is becoming increasingly prominent, opening up a new dimension for the progress of sensing technology.^[^ ^100^ ^]^

As an emerging class of porous materials, MOFs provide unprecedented flexibility in the design of sensing materials due to their extremely high porosity, adjustable pore size, and customizable chemical structure. These characteristics enable MOFs to efficiently capture and identify a wide range of gas molecules, ions, and biomolecules, thus showing great potential for applications in gas sensing, environmental monitoring, and biomedical diagnostics. At the same time, 2D materials, especially GO and MXene, are ideal for building high‐performance sensors due to their excellent electrical conductivity, high chemical stability, and unique 2D structure. Not only do they act as efficient conductive channels, but they also facilitate the rapid transfer of electrical charge. It can also be used as a signal converter to accurately convert chemical or biological signals into measurable electrical signals, significantly improving the sensor's response speed and sensitivity.

By ingeniously integrating MOFs and 2D materials into the polymer matrix, functional polymer composites with high selectivity and high sensitivity can be prepared through the optimization of intermolecular interactions and synergistic effects. These composites not only broaden the range of applications for sensing technology but also provide strong material support for the development of a new generation of high‐performance, multifunctional sensors. It further promotes the in‐depth application and technological innovation of sensing technology in the fields of intelligent manufacturing, environmental monitoring, medical and health care.

#### 4.2.1 Application of MOFs and GO Integration in Sensing

GO is made up of carbon atoms tightly packed to form a hexagonal honeycomb structure, which has excellent electrical, mechanical, and optical properties, making it an ideal material for building high‐performance sensors. In sensing applications, GO's high conductivity and large specific surface area allow it to respond quickly to environmental changes for efficient and sensitive signal conversion. The 2D structure of GO provides abundant surface sites that facilitate interaction with target molecules, thereby improving the specificity and sensitivity of the assay. In addition, GO also has good flexibility and wear resistance, which allows the sensor to maintain stable performance in a variety of harsh environments.

The application of GO in the field of biomedical sensing is particularly prominent. GO‐based biosensors can achieve highly sensitive detection of biomolecules, such as glucose and tumor markers, providing strong support for the early diagnosis and treatment of diseases. At the same time, the excellent biocompatibility of GO makes it an ideal material for the construction of wearable biosensors, which can realize real‐time monitoring of human physiological indicators. In addition, GO is also widely used in gas sensing, photoelectrochemical sensing and flexible pressure sensing. GO‐based gas sensors enable fast and accurate detection of harmful gases, providing safety for environmental monitoring and industrial production. GO photoelectrochemical sensors use the photoelectric effect of GO to achieve efficient conversion and detection of optical signals. With its excellent flexibility and abrasion resistance, GO flexible pressure sensors have shown great application potential in wearable devices, electronic skin, and other fields.

The integration of MOFs and GO enables efficient capture and sensitive detection of target molecules. The porous structure of MOFs provides abundant adsorption sites for molecules in gases and liquids, while the high conductivity of GO enables rapid response and signal conversion to adsorbed molecules. This integrated material offers significant advantages in areas such as gas, biosensing, and photoelectrochemical sensing. Over the past five years, the main research direction of this integration has been focused on electrochemical sensing and gas sensing applications. It is worth noting that these areas have attracted a great deal of attention and have maintained a high level of popularity among researchers.

**Figure 16:**
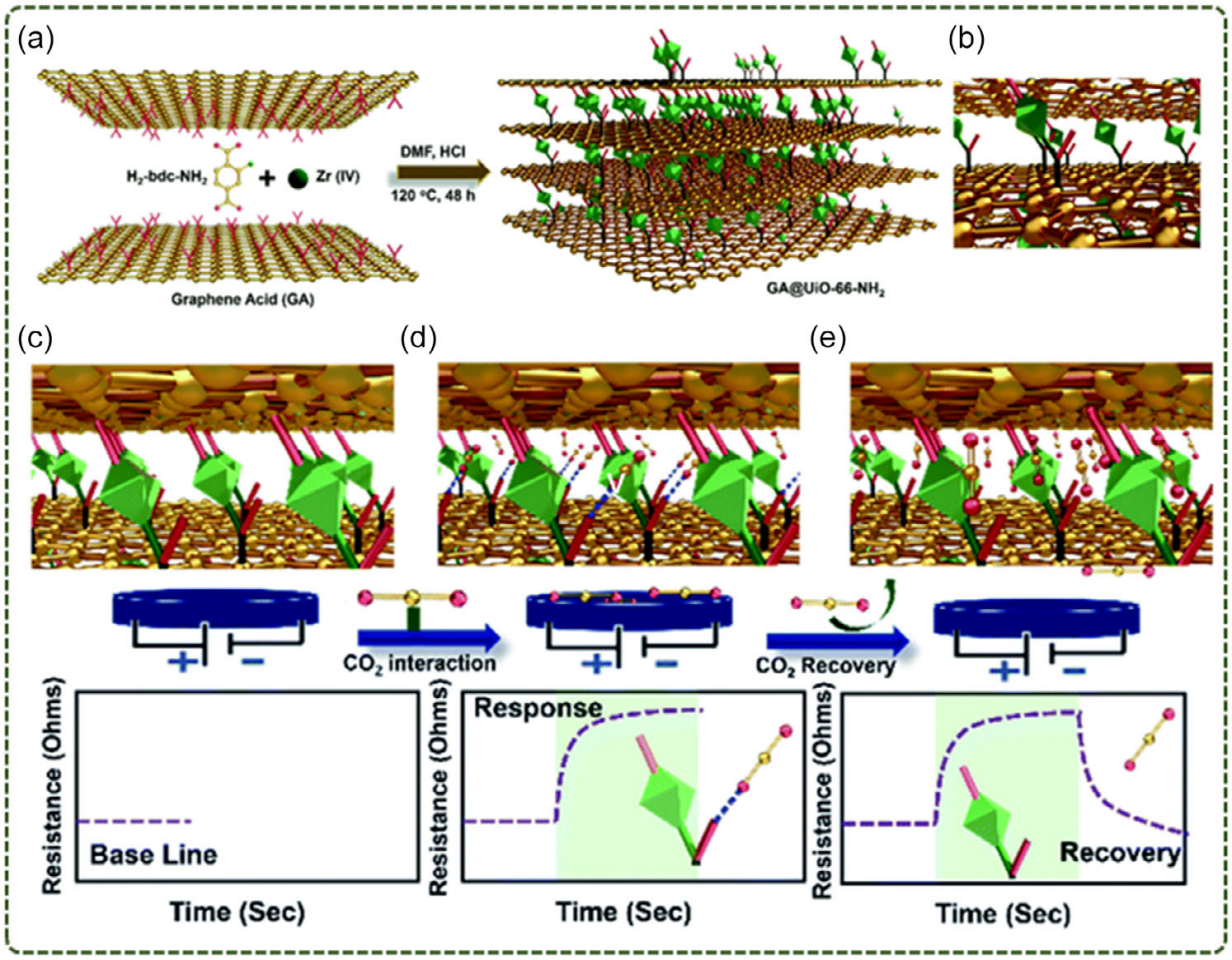
a) Schematic representation of the multifunctional (hierarchical porosity, conductive network, amine/amide functional groups) graphene‐MOF hybrid; b) where carboxyl groups of graphene acid are covalently linked via amide bonds to the amine groups of UiO‐66‐NH_2_ MOF; Schematic illustration of possible sensing mechanism: c) GA@UiO‐66‐NH_2_ shows porous network with amide linkage; d) the CO_2_ interactions with amide linkage present at the basal plane of the GA could bring about the positive change in resistance of GA@UiO‐66‐NH_2_ chemiresistive sensor. e) Upon removal of CO_2_, the steady state baseline has been retrieved suggesting that CO_2_ interaction is reversible (Reproduced (Adapted) with permission. Copyright 2021, Royal Society of Chemistry).

Jayaramulu et al.^[^ ^101^ ^]^ synthesized a GA@UiO‐66‐NH_2_ hybrid by amide bonding using graphenoic acid (GA) and UiO‐66‐NH_2_ as covalent groups (**Figure** **16**a,b). The hybrid material shows layered pores, in which the micropores are located on the octahedral of the UiO‐66‐NH_2_ nanocrystals, while the mesopores are formed by GA stacking of MOF nanocrystals. The layered structure facilitates the diffusion of gases in the material, providing faster interaction sites for the analytes. The GA@UiO‐66‐NH_2_ hybrid provides an interaction site at the amide bond at the interface between GO and MOF for detecting changes in resistance upon CO_2_ interaction (Figure 16c–e). On the one hand, the MOF controls the distance between the conductive GA layers to construct the mesoporous diffusion channel, and on the other hand, it provides the interaction site of the amide functional groups at the GA–MOF interface.

**Figure 17:**
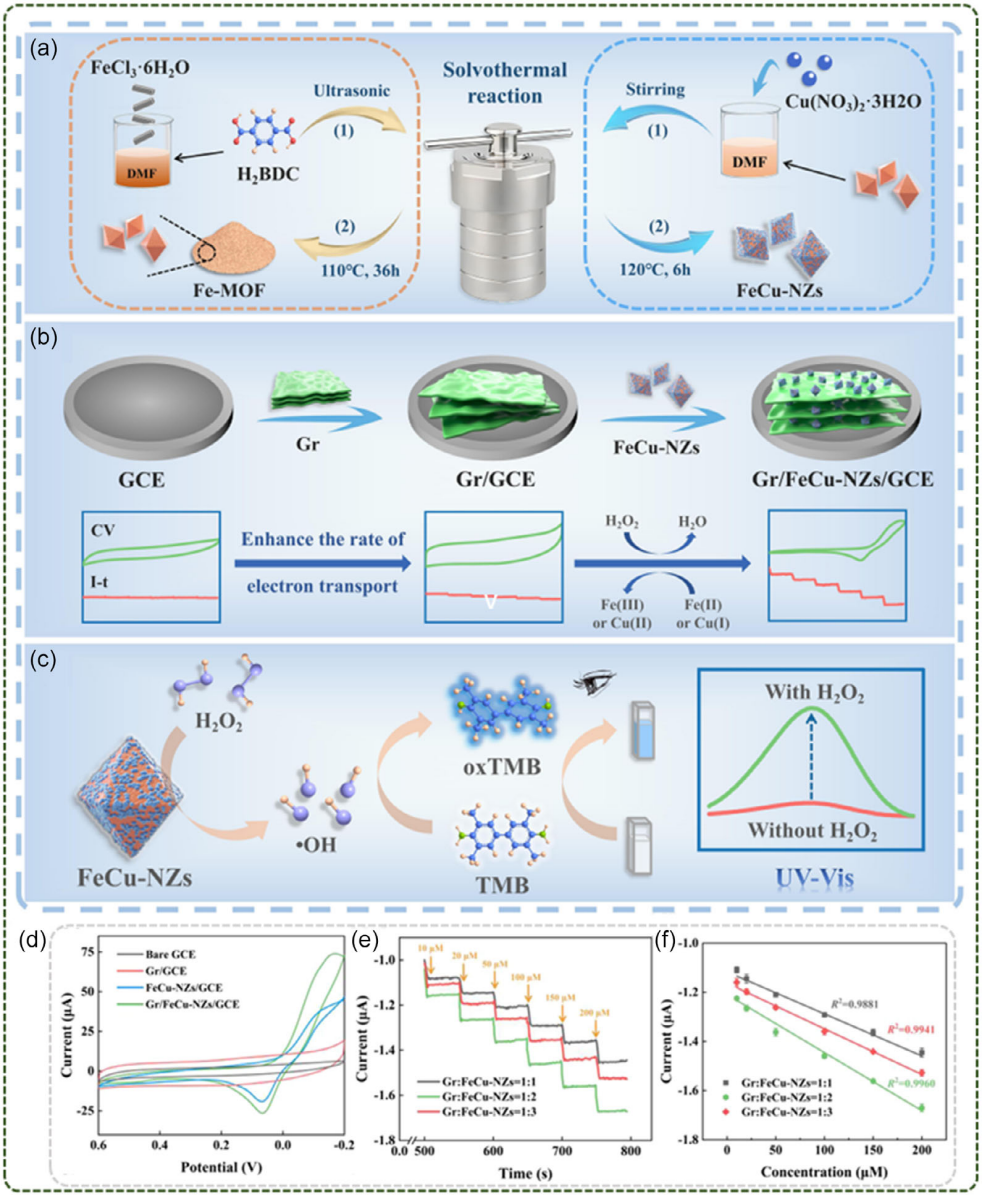
a) Preparation diagram of FeCu‐NZs; b) flow chart of H_2_O_2_ detection by electrochemical; c) colorimetry methods; electrochemical sensing behavior investigation of the Gr/FeCu‐NZs/GCE: d) comparison of the CVs of different modified GCEs in PBS solution containing H_2_O_2_; e) *I*–*t* curves of the fabricated electrode with different Gr/FeCu‐NZs ratios in the range of 10–200 μM and f) the corresponding calibration curves (Reproduced (Adapted) with permission. Copyright 2024, Springer Nature).

Gao et al.^[^ ^102^ ^]^ exploited the synergistic effect of GO and MOF‐on‐MOF nanozymes to develop an electrochemical sensor for the detection of hydrogen peroxide (H_2_O_2_). The preparation process is shown in **Figure** **17**a. First, Fe‐MOF with peroxide‐like activity was synthesized. Subsequently, organic ligands on its surface bind to Cu^2+^ to further enhance enzymatic‐like activity (FeCu‐NZs). Characterization of the materials by cyclic voltammetry and electrochemical impedance spectroscopy showed that the Gr/FeCu‐NZs‐modified GCE electrodes had the highest current response due to the synergistic effect between Gr and FeCu‐NZs (Figure 17b,c). In addition, the introduction of FeCu‐NZs can realize the response current signal of the sensor to H_2_O_2_, and too much FeCu‐NZs will reduce the conductivity of the composite material, thereby reducing the sensitivity. Integration with GO can significantly amplify the electrochemical signal and effectively reduce the detection limit of the sensor (Figure 17d–f).

#### 4.2.2 Integration of MOFs and MXene in Sensing Applications

Thanks to its high electrical conductivity, MXene is able to respond quickly to environmental changes for efficient and sensitive signal conversion. At the same time, its abundant surface functional groups endow MXene with good hydrophilicity, allowing the sensor to interact with the target molecule more effectively, improving the specificity and sensitivity of the detection. In addition, MXene's stable structure enables it to maintain excellent performance in a variety of harsh environments, such as strong acid, strong alkali, high humidity, high salt, and high temperature, which further broadens its application range in the sensing field. MXene is particularly used in the field of gas sensing. By manipulating the layer spacing and surface functional groups of the MXene, selective detection of specific gases can be achieved. For example, MXene‐based gas sensors exhibit excellent sensitivity and versatility in detecting harmful gases such as carbon monoxide, nitrogen dioxide, and ammonia. In addition, MXene is also used in strain sensors, surface‐enhanced Raman scattering sensors, and other fields to achieve accurate detection of physical variables and chemical reactions.

The integration of MOFs and MXene shows significant advantages. For example, Zn‐Co MOFs@MXene composites are used in flexible solid‐state supercapacitors, exhibiting excellent electrochemical properties and mechanical stability. It provides a new idea for the self‐powered energy sensing system of wearable electronic devices. In addition, the integration of MOFs and MXene is also applied to the study of broadband nonlinear optical properties and pulsed laser modulation, revealing their application potential in pulsed light modulators. In the field of biosensing, the integration of MOFs and MXene also shows unique advantages. MOFs can be used as loading and catalytic elements, and combined with the high conductivity of MXene, biosensors with high sensitivity and stability can be constructed. These sensors have a wide range of application prospects in clinical testing, food quality control, environmental monitoring, and other fields. Consistent with the results of the integration of MOFs and GO, its main research areas are also focused on electrochemical sensing and gas sensing. Therefore, this subsection focuses on the literature review of the integration of MOFs with MXene in electrochemical sensing and gas sensing.

**Figure 18:**
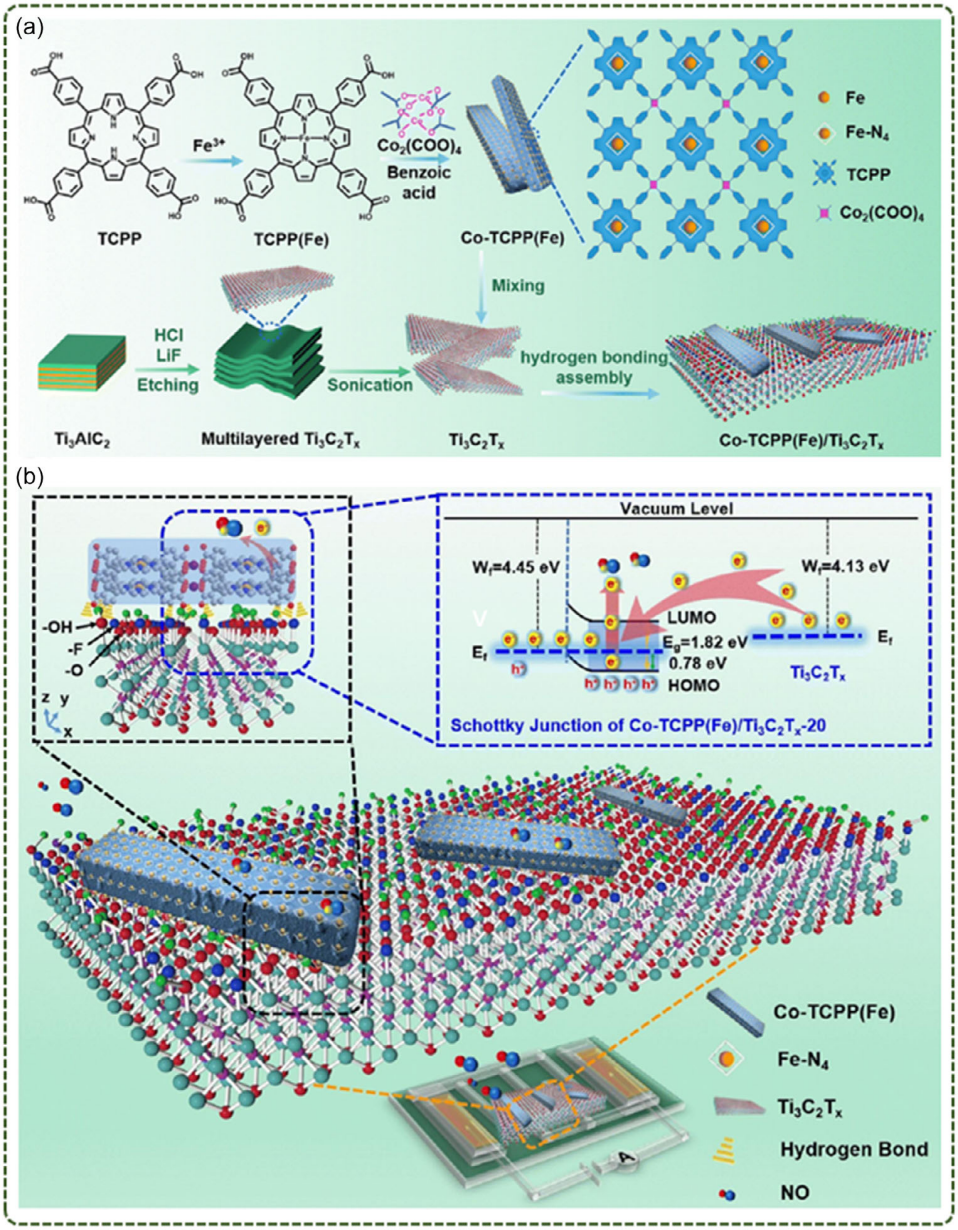
a) Preparation of Co‐TCPP(Fe), Ti_3_C_2_T_*x*_, and Co‐TCPP(Fe)/Ti_3_C_2_T_*x*_ complexes; b) NO sensing principles of Co‐TCPP(Fe)/Ti_3_C_2_T_*x*_‐20 (Reproduced (Adapted) with permission. Copyright 2024, Elsevier).

NO monitoring helps to track the respiratory diseases that patients may have and has important applications in the medical and health field. At present, monitoring NO at the ppb level is a major challenge. Wang et al.^[^ ^74^ ^]^ prepared a rod‐like porphyrin‐based MOF structure by a two‐step method and linked it to MXene by hydrogen bonding to generate a chemically resistant hybrid compound for NO sensing. The preparation process is shown in **Figure** **18**a. This is the first time that the ironporphyrin derivative TCPP(Fe) was coordinated with Co^2+^ to prepare a porphyrin‐based MOF with rod‐like structure. Then, Co‐TCPP(Fe)/MXene was integrated with MXene to obtain hybrid materials. Co‐TCPP(Fe) has a Fermi level (Ef) close to that of its LUMO, proving its n‐type semiconductor properties. Co‐TCPP(Fe)/Ti_3_C_2_T_*x*_‐20 possesses an Ef close to that of the HOMO, demonstrating its p‐type semiconductor properties. Figure 18b shows the NO sensing mechanism based on the Co‐TCPP(Fe)/Ti_3_C_2_T_*x*_‐20 sensor. Co‐TCPP(Fe) and Ti_3_C_2_T_*x*_ form a Schottky junction, which significantly promotes the charge transfer from Co‐TCPP(Fe)/Ti_3_C_2_T_*x*_ to NO in the process of integrated sensing.

In addition, Wang et al.^[^ ^103^ ^]^ reported the construction of 2D heterostructures and 2D MOFs of MXene. First, TCPP was attached to the MXene surface by carboxyl and metal Ti coordination, and then the MXene@MOF heterojunction was synthesized by solvothermal method. Its excellent and stable optoelectronic properties are highly compatible with the requirements of surface plasmon resonance interface sensitizer layer materials. Therefore, MXene@MOF 2D heterojunctions with double gain of optoelectronic properties can be used as SPR‐enhanced interface materials. Through experiments combined with simulation calculations, the plasma electric field and electron transfer at the sensing interface are MXene@MOF enhanced, and the RI sensitivity, detection accuracy and figure of merit of the sensor are improved. In this work, the 2D MOF outer protective layer gives MXene excellent oxidation resistance and photoelectric stability. In addition, MXene@MOF heterojunctions with photoelectric double gain can be used as optoelectronic interface materials for a variety of applications.

## 5 Challenges and Limitations

Although the integration of MOFs with 2D materials shows great application potential in functional polymer composites. But there are still some challenges and limitations to overcome. It is imperative to critically address the prevailing issues in order to facilitate effective design and mitigate practical application problems. Based on existing research, the challenges associated with the development of MOFs‐2D material integrations within functional polymer composites are summarized as follows: 1) The same MOFs are integrated with different 2D materials, and the efficiency of flame retardant and sensing of polymer materials is different. Therefore, this work summarizes the flame retardant and sensing effects and properties of several typical MOFs and 2D materials for different polymers, which provides a valuable reference for future research. 2) As synergists of MOFs, 2D materials can significantly improve the comprehensive properties of composite materials and alleviate the problem of high cost of MOFs. However, the complex and energy‐intensive stripping method of 2D materials is still an urgent problem to be solved. 3) The difficulty of large‐scale production is also an important factor restricting the development of this field. At present, the preparation and compounding process of MOFs and 2D materials is still in the laboratory stage, and it is difficult to achieve large‐scale production. Therefore, new preparation processes and equipment need to be developed to improve production efficiency and reduce costs. 4) The long‐term stability of MOFs and 2D materials in polymer composites directly affects the performance and service life of composite materials. Therefore, long‐term stability assessment studies of the system are needed to understand the performance changes and failure mechanisms of composite materials.

Therefore, optimizing the preparation process and production cost of MOFs and 2D materials is the primary issue, and it is also a necessary prerequisite to expand their large‐scale applications.

## 6 Conclusions and Outlook

In this article, we systematically review the research progress of the integration of MOFs with 2D materials and their application in the field of functional polymer materials. The synthesis strategies, structural characteristics, and diverse application fields of various hybrid materials are summarized in detail. It is clearly pointed out that the hybrid system integrated with MOFs and 2D materials has shown remarkable results in enhancing the versatility of polymer materials. This article conducts an in‐depth and systematic review and summary of the research results of the integration of MOFs and 2D materials in the two key fields of flame‐retardant performance improvement and sensing technology innovation. It aims to provide valuable reference and guidance for subsequent scientific research and practical application in related fields.

MOFs, as a potential carbon source rich in transition metals, exhibit many advantages of highly effective flame retardants. During polymer combustion, transition metals can significantly enhance the density of the cross‐linked network, leading to the formation of a continuous and strong char layer. By blocking the transfer of heat, the dual effects of flame retardant and smoke suppression are realized. The nitrogen oxides released by the pyrolysis of organic ligands are able to dilute the concentration of flammable gases. At the same time, the unique pore structure of MOFs has excellent adsorption capacity for volatile products, which achieves remarkable results in vapor phase flame retardancy. In addition, MOFs demonstrate a wide range of capabilities, from sensing to energy storage. Its unique optoelectronic and semiconductor properties lay a solid foundation for the construction of MOFs‐based sensing interfaces, which can achieve ultrasensitive, highly selective and fast‐response detection of target analytes. 2D materials such as GO and MXene are attracting attention due to their high charge transfer rates. The combination with MOFs not only effectively inhibits the re‐agglomeration of 2D materials but also provides more abundant active sites for the reaction. By uniformly dispersing MOFs particles on the surface of 2D materials and constructing nanocomposites, the dispersion performance of MOFs in polymer matrices can be significantly improved. It can exert a significant synergistic effect, thereby helping to comprehensively improve the comprehensive performance of polymer composites.

Although the integration of MOFs and 2D materials in polymer materials has been widely studied. However, there are still many challenges, and there is still a long way to go for its commercialization and complete replacement of traditional fillers. MOFs and 2D materials continue to attract a lot of attention due to their significant advantages such as large specific surface area, high porosity, and high tunability of structure and properties. With the continuous progress and innovation of science and technology, we have reason to believe that the integration of MOFs and 2D materials will play an indispensable role in a wider range of fields.

## Conflict of Interest

The authors declare no conflict of interest.

## Author Contributions

**Xue Bi**: conceptualization (lead); data curation (lead); formal analysis (lead); investigation (lead); methodology (lead); visualization (lead); writing—original draft (lead); writing—review & editing (lead). **Yanan Hou**: conceptualization (equal); supervision (equal). **Ye‐Tang Pan**: funding acquisition (lead); resources (lead); supervision (lead); validation (lead). **Siqi Huo**: supervision (equal); validation (equal). **Congling Shi**: conceptualization (equal). **Jiyu He**: funding acquisition (equal); project administration (equal); supervision (equal); validation (equal); writing—review & editing (equal). **Rongjie Yang**: funding acquisition (equal); resources (equal); supervision (equal); validation (equal).
